# *Arabidopsis AIP1-2* restricted by *WER*-mediated patterning modulates planar polarity

**DOI:** 10.1242/dev.111013

**Published:** 2015-01-01

**Authors:** Christian S. Kiefer, Andrea R. Claes, Jean-Claude Nzayisenga, Stefano Pietra, Thomas Stanislas, Anke Hüser, Yoshihisa Ikeda, Markus Grebe

**Affiliations:** 1 Umeå Plant Science Centre, Department of Plant Physiology, Umeå University, Umeå SE-90 187, Sweden; 2 Institute of Biochemistry and Biology, Plant Physiology, University of Potsdam, Karl-Liebknecht-Str. 24-25, Building 20, Potsdam-Golm D-14476, Germany

**Keywords:** AIP1, *Arabidopsis*, WEREWOLF, Actin, Patterning, Planar polarity

## Abstract

The coordination of cell polarity within the plane of the tissue layer (planar polarity) is crucial for the development of diverse multicellular organisms. Small Rac/Rho-family GTPases and the actin cytoskeleton contribute to planar polarity formation at sites of polarity establishment in animals and plants. Yet, upstream pathways coordinating planar polarity differ strikingly between kingdoms. In the root of *Arabidopsis thaliana*, a concentration gradient of the phytohormone auxin coordinates polar recruitment of Rho-of-plant (ROP) to sites of polar epidermal hair initiation. However, little is known about cytoskeletal components and interactions that contribute to this planar polarity or about their relation to the patterning machinery. Here, we show that ACTIN7 (ACT7) represents a main actin isoform required for planar polarity of root hair positioning, interacting with the negative modulator ACTIN-INTERACTING PROTEIN1-2 (AIP1-2). *ACT7*, *AIP1-2* and their genetic interaction are required for coordinated planar polarity of ROP downstream of ethylene signalling. Strikingly, AIP1-2 displays hair cell file-enriched expression, restricted by *WEREWOLF* (*WER*)-dependent patterning and modified by ethylene and auxin action. Hence, our findings reveal AIP1-2, expressed under control of the *WER-*dependent patterning machinery and the ethylene signalling pathway, as a modulator of actin-mediated planar polarity.

## Abstract (precis)

**Summary**: Planar polarity in *Arabidopsis* is shaped by ACTIN-INTERACTING PROTEIN1-2, which is under the control of WEREWOLF-dependent patterning and ethylene signalling.

## INTRODUCTION

Development of multicellular organisms relies on formation and maintenance of structural asymmetries at the single-cell level as well as their coordination within the tissue context. Whereas cell polarity describes axially organised asymmetries in subcellular structures or intracellular molecule distributions (Yang, 2008), planar polarity defines the coordinated organisation of cells within a plane of a tissue layer (Nübler-Jung, 1987). The polar orientation of hairs in the *Drosophila* wing epithelium is widely used as a model system for planar polarity in animals (Adler, 2002). Similarly, the emergence of root hairs in the *Arabidopsis* root epidermis represents a model system to unveil mechanisms underlying planar polarity in plants (Nakamura et al., 2012). The root epidermis is composed of alternating files of hair-forming cells (trichoblasts) and non-hair-forming cells (atrichoblasts), the fate of which is specified by the WEREWOLF (WER) MYB transcription factor-dependent patterning system (Galway et al., 1994; Masucci et al., 1996; Lee and Schiefelbein, 1999). Root hair-forming cells develop root hairs as tubular protrusions from their outer membrane, where hairs are uniformly initiated towards, albeit not completely at, the root tip-oriented (basal) ends of cells (Masucci and Schiefelbein, 1994). In contrast to *Drosophila*, in which the long-range polarising cue instructing the Frizzled planar cell polarity pathway is provided by Wingless and its homologue dWnt4 (Wu et al., 2013), vectorial information for planar polarity in *Arabidopsis* is provided by a concentration gradient of the phytohormone auxin (Fischer et al., 2006; Ikeda et al., 2009). Formation of this gradient depends on local auxin biosynthesis in the root tip, where auxin concentration reaches its maximum, and on the basipetal (shootward) transport of auxin in the root epidermis (Ikeda et al., 2009). Local upregulation of auxin biosynthesis induced by mutations in the *CONSTITUTIVE TRIPLE RESPONSE1* (*CTR1*) gene, a repressor of the auxin gradient, causes hyperpolar positioning of root hairs at basal-most ends of cells (Ikeda et al., 2009). In analogy to animal systems, in which Rac/Rho-family GTPases are downstream effectors of planar cell polarity (Goodrich and Strutt, 2011), auxin instructs polar accumulation of Rho-of-plant (ROP) GTPases at the outer plasma membrane (Fischer et al., 2006; Ikeda et al., 2009), thus marking the root hair initiation site (Molendijk et al., 2001; Jones et al., 2002). During planar morphogenesis in the leaf epidermis, ROP2 and ROP4 regulate actin organisation, whereas ROP6 modulates the microtubule cytoskeleton (Fu et al., 2009; Xu et al., 2010). In the root epidermis, ROP6 is thought to contribute to actin organisation (Lin et al., 2012). Among the eight expressed actin paralogues encoded in the *Arabidopsis* genome, *ACT2*, *ACT7* and *ACT8*, and potentially also *ACT11* (Cvrčková et al., 2010), contribute to root development (Kandasamy et al., 2009). Mutant alleles of *ACT2* display weak defects in root hair positioning (Ringli et al., 2002), but mechanisms regulating the actin cytoskeleton during planar polarity formation in plants remain largely unknown. In *Drosophila*, the single actin-depolymerizing factor (ADF) Twinstar (Tsr) interacts with the single actin-interacting protein 1 (AIP1) Flare (Flr) during epithelial morphogenesis (Chu et al., 2012). Both negatively regulate actin filament organisation required for planar cell polarity downstream of the Frizzled pathway (Blair et al., 2006; Ren et al., 2007). AIP1 homologues from several systems, such as Aip1p from yeast and AIP1-1 from *Arabidopsis*, have been shown to enhance the F-actin depolymerizing activity of ADF (named Cofilin in yeast) *in vitro* (Rodal et al., 1999; Allwood et al., 2002). In *Arabidopsis*, simultaneous downregulation of the reproductive isoform *AIP1-1* and the vegetative isoform *AIP1-2* by RNA interference (RNAi), as well as ectopic overexpression of *AIP1-1*, affect actin organisation and root hair tip growth (Ketelaar et al., 2004, 2007). Yet, analyses of individual functions of the *Arabidopsis AIP1* genes are lacking. Here, we report that *AIP1-2* and *ACT7* interact and are required for polar root hair positioning downstream of *CTR1*. We demonstrate that *WEREWOLF*-dependent patterning specifies AIP1-2 expression, suggesting that *AIP1-2* function during auxin-mediated planar polarity becomes spatially restricted by cell fate patterning.

## RESULTS

### *ACT2* and *ACT7* are required for planar polarity downstream of *CTR1*

**Fig. 1.**
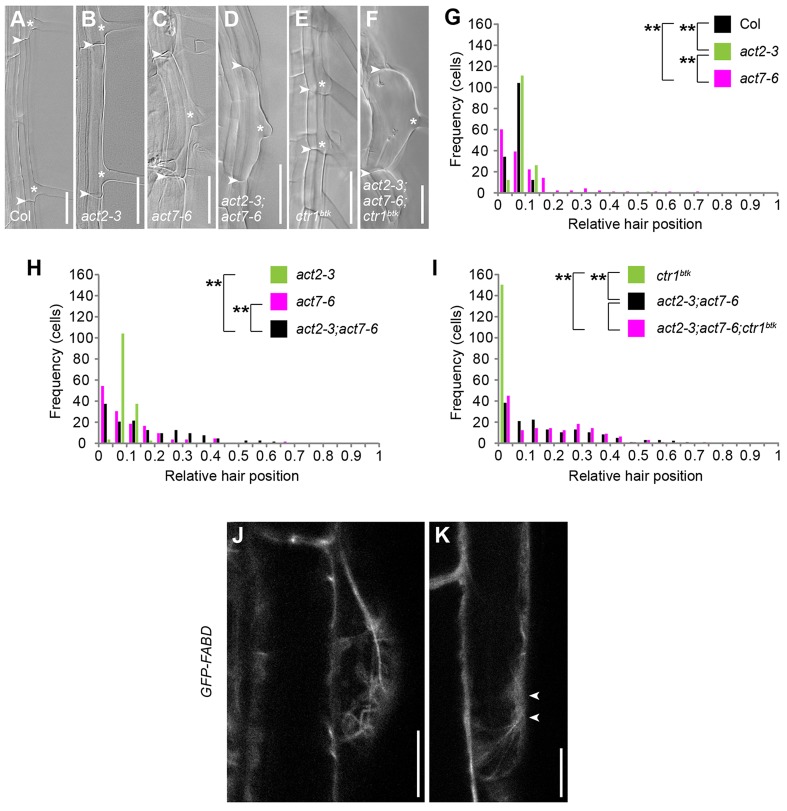
***ACT2* and *ACT7* are required for planar polarity formation downstream of *CTR1*.** (A-F) Root hair-forming cells of five-day-old (A) wild-type Columbia (Col), (B) *act2-3*, (C) *act7-6*, (D) *act2-3;act7-6*, (E) *ctr1^btk^* and (F) *act2-3;act7-6;ctr1^btk^* seedlings. Arrowheads mark apical and basal ends of cells; asterisks mark hair initiation sites. (G-I) Quantitative analysis of root hair-positioning phenotypes of lines depicted in A-F, displaying number of cells (frequency) with relative hair positions in classes from 0 (basal-most) to 1 (apical-most). *n*=150 cells from 30 roots per genotype. Significance *P* values were determined by non-parametric, two-sample Kolmogorov–Smirnov (KS) test. (G) **P=0.000 Col versus *act2-3*; **P=0.000 Col versus *act7-6*; **P=0.000 *act2-3* versus *act7-6*. (H) **P=0.000 *act2-3* versus *act2-3;act7-6*; **P=0.003 *act7-6* versus *act2-3;act7-6*. (I) **P=0.000 *ctr1^btk^* versus *act2-3;act7-6*; **P=0.000 *ctr1^btk^* versus *act2-3;act7-6;ctr1^btk^*; *P*=0.706 *act2-3;act7-6* versus *act2-3;act7-6;ctr1^btk^*. (J,K) Confocal laser scanning microscopy (CLSM) image projections of GFP-FABD-labelled actin filaments (J) in a forming root hair bulge and (K) at hair initiation site prior to bulging. Arrowheads in K indicate site of future hair emergence. Scale bars: 50 µm in A-F; 10 µm in J,K.

To further investigate the role of actin during planar polarity formation, we analysed root hair position phenotypes of *act2*, *act7* and *act8* loss-of-function mutants. We employed the *act2-3* null allele (Nishimura et al., 2003) and an *act7* T-DNA line with an insertion in the first exon (SALK_131610) that shows a twofold reduction of total actin levels (Guo et al., 2013), which we refer to as *act7-6*. In addition, we employed an *act7* T-DNA line carrying a single insertion in the third exon of *ACT7* (GK-498G06), which we named *act7-7*, as well as *act8-2* (Kandasamy et al., 2009). We found that root hair position shifted slightly apically in *act2-3* when compared with wild type (WT) (Fig. 1A,B,G). More strikingly, hair positions in *act7-6* and *act7-7* were widely distributed along the apical-basal axis of cells, revealing both an apical and a basal shift (Fig. 1A,C,G; supplementary material Fig. S1A,B). We established allelism between *act7-6* and *act7-7* by analysing the *act7-6/act7-7* (*act7^-6/-7^*) trans-heterozygous mutant, which was indistinguishable from *act7-6* and *act7-7* homozygotes (supplementary material Fig. S1A-C). In comparison, the root hair position phenotype of *act8-2* did not differ from WT (supplementary material Fig. S1D). Defects in polar hair positioning were significantly stronger in *act7* compared with the *act2* allele (Fig. 1B,C,G; supplementary material Fig. S1A,B) and much more pronounced in the *act2-3;act7-6* double mutant when compared with the single mutants (Fig. 1B-D,H), suggesting that *ACT7* contributes more strongly to planar polarity than *ACT2*, but that both synergistically act on polar root hair positioning.

We next addressed the genetic relationship between *ACT2*, *ACT7* and *CTR1*. Whereas *act7-6;ctr1^btk^* and *act2-3;ctr1^btk^* double mutants revealed partial suppression of the hyperpolar *ctr1^btk^* root hair-positioning phenotype (supplementary material Fig. S1E,F), the *act2-3;act7-6;ctr1^btk^* triple mutant displayed root hair placement indistinguishable from the *act2-3;act7-6* double mutant (Fig. 1D,F,I), thus revealing full suppression of the *ctr1^btk^* effect on polar hair placement (Fig. 1D-F,I). This demonstrates the requirement of *ACT2* and *ACT7* for planar polarity downstream of *CTR1*.

To study the distribution of filamentous actin (F-actin) during polar hair positioning, we visualised actin during early stages of root hair formation using the actin-tubulin double-marker line *GFP-FABD;mCherry-TUA5*. We observed a dense actin network in forming root hair bulges (Fig. 1J), as reported previously (Baluska et al., 2000). In addition, we identified actin filaments in vicinity of the future hair initiation site prior to bulge formation (Fig. 1K), supporting a role for actin in polar site selection. Similar results were obtained using the *GFP-ABD2-GFP* and *Lifeact-Venus* markers or the F-actin-binding probe BODIPY FL phallacidin (supplementary material Fig. S1G-J). However, we did not observe a significant difference in actin cytoskeleton organisation in the basal region of trichoblasts when compared with the apical ends of the same cells (supplementary material Fig. S1K-M). Our findings reveal that planar polarity strongly depends on *ACT7*, which synergistically acts with *ACT2* during selection of the polar hair initiation site downstream of *CTR1*. We also observed that F-actin is present during hair initiation, although not particularly enriched at the hair initiation site.

### *ACT7* interacts with *AIP1-2* in yeast and *in vitro*

**Fig. 2.**
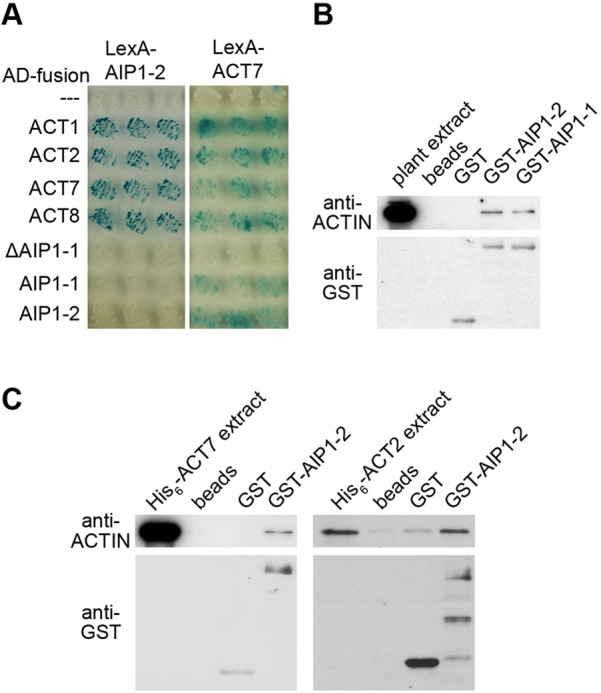
**AIP1-2 interaction with *Arabidopsis* ACTINs in yeast and *in vitro*.** (A) LexA-AIP1-2 interaction with B42 transcriptional activation domain (AD)-fusions of ACT1, ACT2, ACT7 and ACT8 (left panel), and LexA-ACT7 interaction with AD-fusions of ACT1, ACT2, ACT7, ACT8, AIP1-1 and AIP1-2 (right panel) in the yeast two-hybrid system. Yeasts were grown for 24 (left panel) and 48 h (right panel). Empty prey vector (---) and truncated AIP1-1 lacking amino acids 1-137 (ΔAIP1-1) are negative controls. (B,C) Empty glutathione sepharose (beads) or beads carrying GST only (GST) are negative controls. Loading of sepharose beads was tested using anti-GST antibody in western blots. (B) Glutathione-sepharose-bound GST-fusions of AIP1-2 (GST-AIP1-2) and AIP1-1 (GST-AIP1-1) precipitate actin from *Arabidopsis* protein extracts. (C) Glutathione-sepharose-bound GST-fusion of AIP1-2 (GST-AIP1-2) precipitates 6×His-fusions of ACT7 (His_6_-ACT7) and ACT2 (His_6_-ACT2) from bacterial protein extracts.

To identify interactors of actin, we conducted a yeast two-hybrid screen of a cDNA library prepared from *Arabidopsis* seedlings using ACT7 as bait, revealing AIP1-2 (At3g18060) as a single interactor. We confirmed the interaction in pairwise yeast two-hybrid assays by further including reproductive ACT1, displaying highest levels of gene expression in pollen (An et al., 1996), ACT2, ACT8 and AIP1-1. All actins strongly interacted with AIP1-1 and AIP1-2 (Fig. 2A; supplementary material Fig. S2A), but not a truncated form of AIP1-1 (ΔAIP1-1), lacking the first 137 amino acids of the protein when either used as bait or prey (supplementary material Fig. S2A-C). We independently evaluated interactions for the two actin isoforms that were of particular interest with respect to planar polarity, ACT2 and ACT7, by glutathione-S-transferase (GST) *in vitro* pull-down assays. Bacterially expressed GST-AIP1-1 or GST-AIP1-2, bound to glutathione sepharose beads, specifically precipitated actin from *Arabidopsis* protein extracts (Fig. 2B; supplementary material Fig. S2D). Furthermore, GST-AIP1-2 precipitated both 6×Histidine-tagged (6×His)-fusions with ACT2 and ACT7 from bacterial protein extracts (Fig. 2C; supplementary material Fig. S2E,F). These results identify AIP1-2 as an interactor of ACT7, ACT2 and other actin isoforms in yeast and *in vitro*.

### *AIP1-2* genetically interacts with *ACT7*

**Fig. 3.**
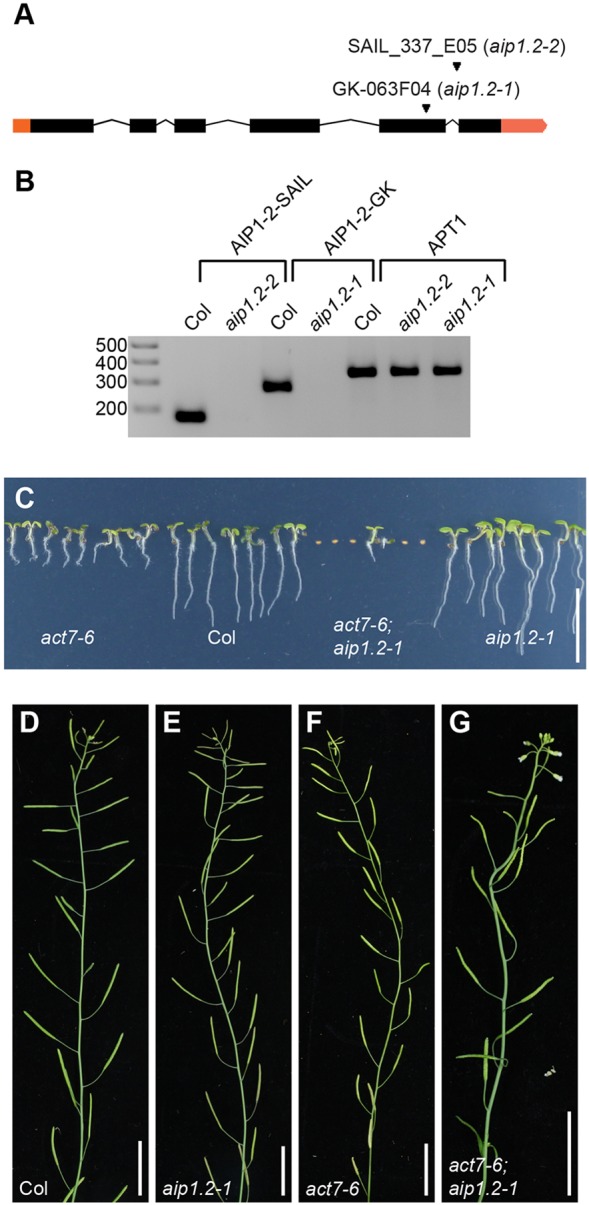
***AIP1-2* and *ACT7* genetically interact in *Arabidopsis*.** (A) *AIP1-2* locus map. Orange boxes, untranslated regions; black boxes, exons. Arrowheads, positions of T-DNA insertions. (B) Semi-quantitative RT-PCR analysis of Col wild-type and *aip1-2* mutant seedlings. Regions surrounding the T-DNA insertion sites were amplified from Col but not from the respective *aip1-2* mutant cDNA. *APT1* was used as a reference gene. (C) Five-day-old *act7-6*, Col, *act7-6;aip1.2-1* and *aip1.2-1* seedlings. Germination of *act7-6;aip1.2-1* seeds is strongly reduced compared with Col, *act7-6* and *aip1.2-1*. (D-G) Main stems of three-week-old Col, *aip1.2-1*, *act7-6* and *act7-6;aip1.2-1* plants. Scale bars: 10 mm in C; 20 mm in D-G.

To address *AIP1-2* function *in planta*, we characterised two lines carrying a T-DNA insertion in the *AIP1-2* gene, GK-063F04 with an insertion in the fifth exon and SAIL_337_E05 with an insertion in the fifth intron, and named them *aip1.2-1* and *aip1.2-2*, respectively (Fig. 3A). Semi-quantitative RT-PCR revealed that neither *aip1.2-1* nor *aip1.2-2* produced a full-length *AIP1-2* transcript (Fig. 3B). To analyse potential genetic interactions between *ACT7* and *AIP1-2*, we generated double mutants between *aip1.2-1* or *aip1.2-2* and *act7-6* or *act7-7*. Whereas WT and single mutants germinated at rates between 89% and 100%, germination of *act7;aip1-2* double mutants was reduced to 14-21% (Table 1), uncovering a synergistic effect of the *aip1-2* and *act7* mutations that resulted in synthetic lethality of most double mutants (Fig. 3C; supplementary material Fig. S3A). This synergistic effect might be due to strong interaction between *ACT7* and *AIP1-2* as well as to additional interactions between *AIP1-2* and *ACT7* with *ACT2* or other actins. As the germination defect was not fully penetrant, some double-mutant seeds eventually germinated and could be used to analyse adult plants with this low-penetrance phenotype. These *act7;aip1-2* double mutants displayed striking alterations of shoot architecture, such as twisted stems and siliques abnormally oriented along the shoot, when compared with the single mutants (Fig. 3D-G; supplementary material Fig. S3B-F). Our results reveal synergistic genetic interaction between *AIP1-2* and *ACT7*, strongly suggesting that the AIP1-2 and ACT7 protein-protein interaction observed in yeast and *in vitro* is relevant during *Arabidopsis* development.

**Table 1.**
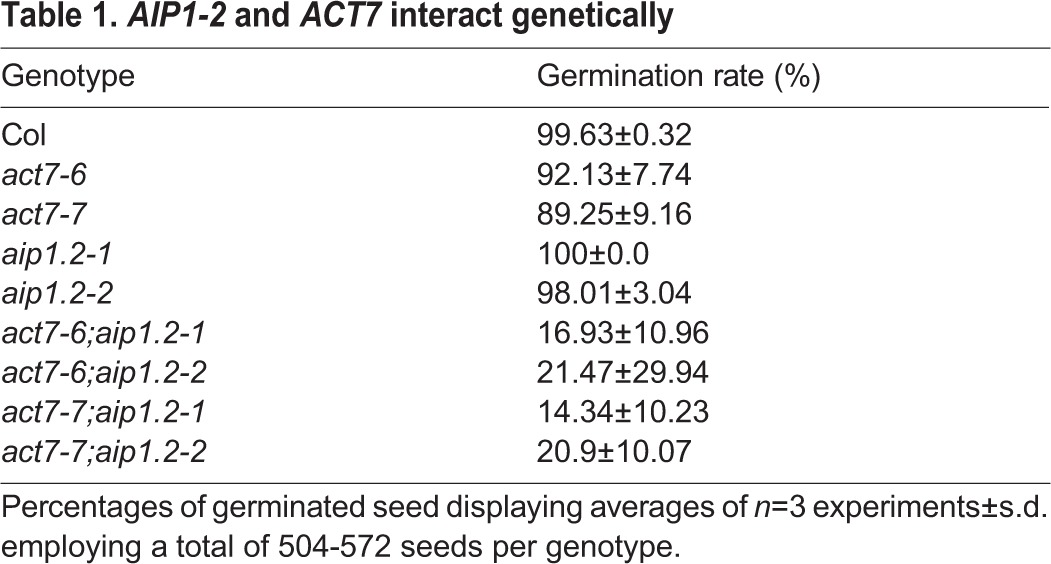
*AIP1-2* and *ACT7* interact genetically

### AIP1-2 is predominantly expressed in hair cell files and contributes to planar polarity

**Fig. 4.**
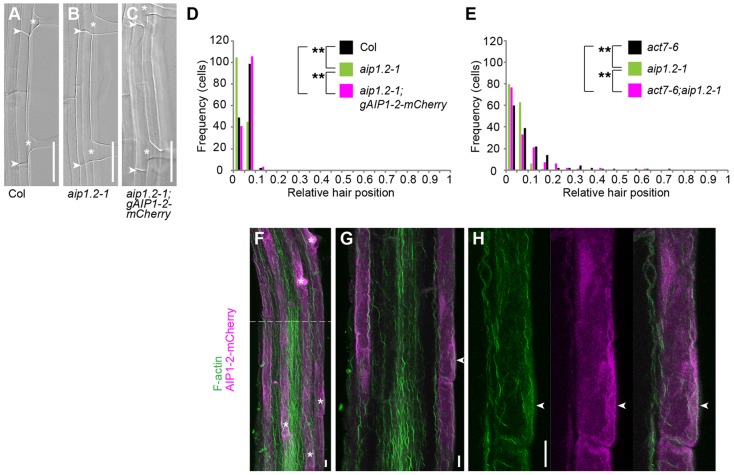
**AIP1-2 modulates planar polarity together with ACT7 and co-localises with actin at root hair initiation sites.** (A-C) Root-hair forming cells of five-day-old (A) wild-type Columbia (Col), (B) *aip1.2-1* and (C) *aip1.2-1;gAIP1-2-mCherry* seedlings. Arrowheads, apical and basal ends of cells; asterisks, root hair initiation sites. (D,E) Quantitative analysis of relative root hair position of (D) *aip1.2-1* and *aip1.2-1;gAIP1-2-mCherry* compared with Col, and (E) *act7-6* and *aip1.2-1* compared with *act7-6;aip1.2-1. n*=150 cells from 30 roots per genotype. Significance *P* values were determined by KS test. (D) **P=0.000 Col versus *aip1.2-1*; *P*=0.097 Col versus *aip1.2-1;gAIP1-2-mCherry*; **P=0.000 *aip1.2-1* versus *aip1.2-1;gAIP1-2-mCherry*. (E) **P=0.000 *act7-6* versus *aip1.2-1*; *P*=0.215 *act7-6* versus *act7-6;aip1.2-1*; **P=0.000 *aip1.2-1* versus *act7-6;aip1.2-1*. (F-H) CLSM images of early root differentiation zone of five-day-old *aip1.2-1;gAIP1-2-mCherry* seedlings probed for F-actin with BODIPY FL phallacidin. (F) Top view displaying AIP1-2-mCherry fluorescence preferentially in hair-forming cell files. Asterisks, root hair initiation sites. (G) Transversal section showing AIP1-2-mCherry and F-actin accumulation at sites of future hair emergence. (H) Magnification of root hair initiation site (arrowheads) shown in G. Scale bars: 50 µm in A-C; 10 µm in F-H.

To address whether *AIP1-2* contributes to planar polarity, we analysed root hair positioning in *aip1-2* mutants. In comparison with the wild type (Fig. 4A), root hair placement was shifted basally in *aip1.2-1* (Fig. 4B,D) and in *aip1.2-2* (supplementary material Fig. S4A). These defects were caused by reduced *AIP1-2* function, because when we reintroduced a C-terminally mCherry-tagged or a 3×Myc-tagged fusion of AIP1-2 expressed under control of *AIP1-2* genomic sequences in the *aip1.2-1* mutant background, *aip1.2-1-*mutant phenotypes were restored to wild-type levels (Fig. 4A-D; supplementary material Fig. S4B).

To further investigate whether *ACT7* interacts with *AIP1-2* during planar polarity establishment, we analysed the phenotypes of *act7;aip1-2* double mutants in which the genetic interaction was not fully penetrant. In these seedlings, root hair position was indistinguishable between *act7-7* single and *act7-7;aip1.2-1* double mutants as well as between four different allele combinations of *act7;aip1-2* double mutants (Fig. 4E; supplementary material Fig. S4C,D), suggesting that *act7* behaves epistatically over *aip1-2* in these plants.

We next examined the localisation of AIP1-2 and actin during root hair initiation. Strikingly, AIP1-2-mCherry was most strongly expressed in the epidermis and specifically enriched in trichoblast cell files (Fig. 4F,G). At the subcellular level, AIP1-2-mCherry co-localised with the cytosolic marker WAVE 1Y except for the additional nuclear localisation of WAVE 1Y (supplementary material Fig. S4E,F). Together with actin, AIP1-2-mCherry was abundant in outgrowing root hair bulges (Fig. 4F) as well as at hair initiation sites (Fig. 4G,H). The abundance of actin at root hair initiation sites did not differ between the wild type and the *aip1.2-1* mutant (supplementary material Fig. S4G,H). Similarly, quantitative evaluation of actin-filament bundling as a measure of actin filament organisation (see methods in the supplementary material) in trichoblasts did not reveal a difference in actin cytoskeleton organisation between *aip1.2-1* and the wild type (supplementary material Fig. S4I). This is consistent with the only subtle defects in polar hair positioning observed in *aip1-2* single mutants, which might be difficult to resolve at the level of actin organisation. Together, our results reveal a function for *AIP1-2* in planar polarity, its genetic interaction with *ACT7*, hair cell file-specific expression of AIP1-2 and its co-localisation with F-actin at sites of root hair initiation. The basal polarity shift observed in *aip1-2* mutants suggests *AIP1-2* as a negative modulator of planar polarity.

### *AIP1-2* and *ACT7* are required for polar ROP positioning

**Fig. 5.**
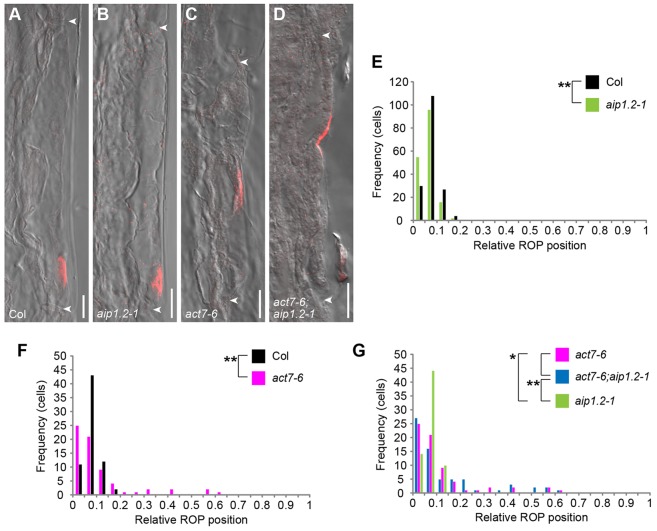
***AIP1-2*, *ACT7* and their interaction mediate polar ROP positioning.** (A-D) Anti-ROP (red) immunofluorescence in (A) Col, (B) *aip1.2-1*, (C) *act7-6* and (D) *act7-6;aip1.2-1*, combined with differential interference contrast (DIC) microscopy image. Arrowheads, apical and basal ends of cells. Scale bars: 10 µm. (E-G) Quantitative analysis of relative ROP positioning of (E) *aip1.2-1* and (F) *act7-6* compared with Col, and of (G) *aip1.2-1* and *act7-6* compared with *act7-6;aip1.2-1*. Number of cells (frequency) with relative apical-basal ROP protein patch position in classes from 0 (basal-most) to 1 (apical-most). *n*=169 for Col and *aip1.2-1*, 68 for *act7-6* and 78 for *act7-6;aip1.2-1*; significance *P* values were determined by KS test. (E) **P=0.006 Col versus *aip1.2-1*. (F) *P=0.014 Col versus *act7-6*. (G) *P*=0.707 *act7-6* versus *act7-6;aip1.2-1*; **P=0.004 *aip1.2-1* versus *act7-6;aip1.2-1*; *P=0.023 *act7-6* versus *aip1.2-1*.

To address whether *AIP1-2* and *ACT7* affect polar positioning of ROP proteins at sites of hair initiation, we raised an antibody against an AtROP2-derived peptide. The affinity-purified antibody (see methods in the supplementary material) recognised a single band of the expected size at 21.5 kDa on western blots from *Arabidopsis* seedling protein extracts (see methods in the supplementary material), and we detected EYFP-ROP2 and GFP-ROP6 in lines expressing these proteins (supplementary material Fig. S5). Moreover, the purified serum marked the hair initiation site, where it co-labelled with EYFP-ROP2 (supplementary material Fig. S5). When compared with the wild type (Fig. 5A,E), polar ROP position was shifted basally in *aip1.2-1* (Fig. 5B,E), whereas ROP patches were shifted basally and apically in *act7-6* (Fig. 5C,F). Furthermore, *act7-6* behaved epistatically over *aip1.2-1*, as the ROP position did not differ between the *act7-6* single and the *act7-6;aip1.2-1* double mutant (Fig. 5C,D,G). However, ROP patch distribution significantly differed between *aip1.2-1* and *act7-6* or *act7-6;aip1.2-1* mutants (Fig. 5B-D,G). These results show that *AIP1-2* and *ACT7* are required for correct polar positioning of ROP proteins during establishment of planar polarity.

### *WEREWOLF* and ethylene signalling mediate patterned AIP1-2 expression

**Fig. 6.**
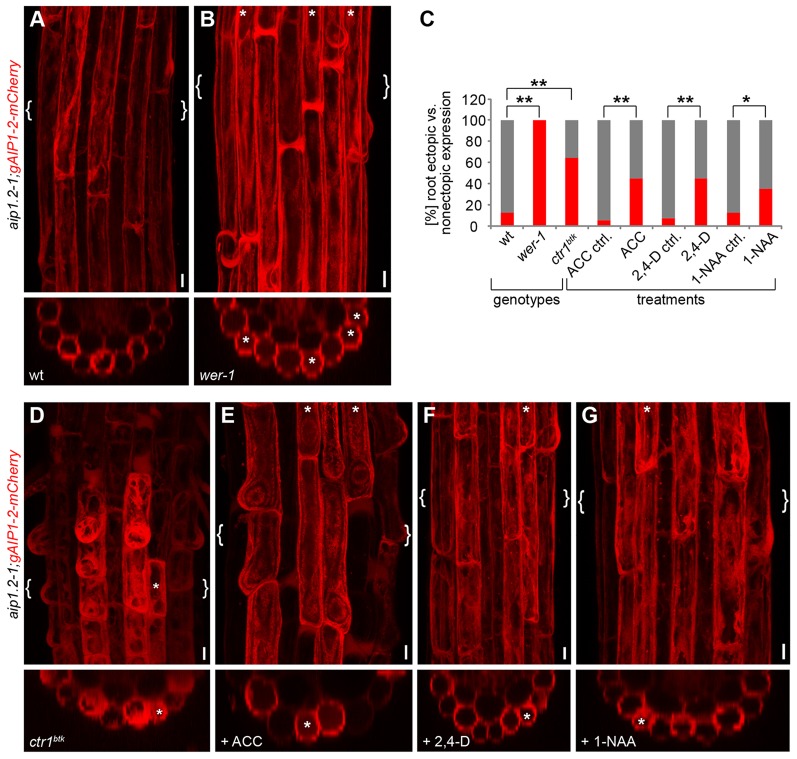
**Hair cell file-specific AIP1-2 expression depends on *WEREWOLF* and ethylene signalling.** (A,B) CLSM image projections displaying AIP1-2-mCherry fluorescence in root differentiation zone of five-day-old (A) *aip1.2-1;gAIP1-2-mCherry* (WT) and (B) *wer-1;aip1.2-1;gAIP1-2-mCherry* (*wer-1*) seedlings. Upper panels are top views and lower panels transversal sections of the regions indicated by curly brackets. Asterisks indicate ectopic AIP1-2-mCherry enrichment in non-hair cell files. (C) Percentage of five-day-old seedling roots displaying strictly root hair file-specific (grey) or additional ectopic (red) AIP1-2-mCherry expression in *aip1.2-1;gAIP1-2-mCherry* (wt; *n*=16 seedlings), *wer-1;aip1.2-1;gAIP1-2-mCherry* (*wer-1*; *n*=15 seedlings), *ctr1^btk^;aip1.2-1;gAIP1-2-mCherry* (*ctr1^btk^*; *n*=14 seedlings) seedlings and *aip1.2-1;gAIP1-2-mCherry* seedlings grown on media supplemented with 5 µM ACC, 20 nM 2,4-D and 100 nM 1-NAA (*n*=40 seedlings per treatment). Significances were determined by Fisher's exact test with significance levels *P<0.05 and **P<0.01. Supplementary material Table S1 shows exact *P* values. (D-G) CLSM projections displaying AIP1-2-mCherry fluorescence in root differentiation zone of five-day-old (D) *ctr1^btk^;aip1.2-1;gAIP1-2-mCherry* (*ctr1^btk^*) or *aip1.2-1;gAIP1-2-mCherry* seedlings grown on media supplemented with (E) 5 µM ACC, (F) 20 nM 2,4-D and (G) 100 nM 1-NAA. Upper panels are top views and lower panels transversal sections of the regions indicated by curly brackets. Asterisks, ectopic AIP1-2-mCherry enrichment in non-hair cell files. Scale bars: 10 µm.

Our findings that AIP1-2 displays hair cell file-enriched expression prompted us to investigate whether its expression relies on WER, a key regulator of cell fate patterning in the root epidermis that controls specification of non-hair cell fate (Lee and Schiefelbein, 1999). Accordingly, most epidermal cells acquire hair cell fate in *wer* loss-of-function mutants, forming ectopic hairs in non-hair positions. Non-hair positions are those in which epidermal cells contact only one cell file of the underlying cortical tissue layer (Dolan et al., 1994). Strikingly, we observed strong AIP1-2-mCherry expression in all epidermal cell files in *wer-1* mutants, (Fig. 6A,B), revealing that *WER* specifies AIP1-2 expression.

Interestingly, both *CTR1*-dependent ethylene signalling and auxin signalling contribute to root hair cell differentiation downstream of cell fate regulation (Dolan et al., 1994; Tanimoto et al., 1995; Masucci and Schiefelbein, 1996). We therefore investigated AIP1-2-mCherry expression in the *ctr1^btk^* mutant or upon application of the ethylene precursor 1-Aminocyclopropane-1-carboxylic acid (ACC) and observed cells forming hairs in non-hair positions that expressed AIP1-2-mCherry in the *ctr1^btk^;aip1.2-1* mutant background (Fig. 6C,D), as well as in *aip1.2-1* seedling roots grown on ACC-containing medium (Fig. 6C,E). These findings revealed the dependence of patterned AIP1-2 expression on *CTR1* function and balanced ethylene levels.

To test whether exogenous application of auxin had a similar effect on AIP1-2 expression, we applied the synthetic auxins 2,4-Dichlorophenoxyacetic acid (2,4-D) or 1-Naphthaleneacetic acid (1-NAA) to the growth medium of AIP1-2-mCherry-expressing seedlings. Strikingly, we observed an increase in ectopic AIP1-2-mCherry expression in non-hair positions in seedlings treated with 2,4-D (Fig. 6C,F) or 1-NAA (Fig. 6C,G). Together, these results demonstrate that cell file-specific AIP1-2 expression depends on *WER* function and ethylene signalling, and that it is sensitive to auxin concentration.

### *CTR1* is epistatic over *AIP1-2* during root hair positioning

**Fig. 7.**
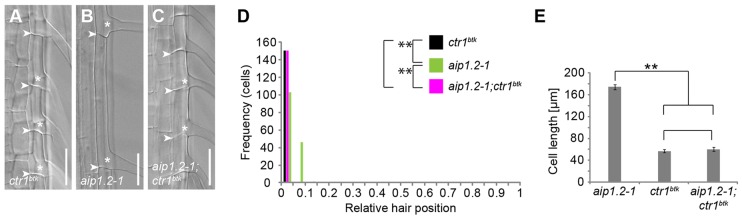
***CTR1* is epistatic over *AIP1-2*.** (A-C) Root-hair forming cells of five-day-old (A) *ctr1^btk^*, (B) *aip1.2-1* and (C) *aip1.2-1;ctr1^btk^* seedlings. Arrowheads, apical and basal ends of cells; asterisks, root hair initiation sites. Scale bars: 50 µm. (D) Quantitative analysis of relative root hair-positioning phenotypes of *ctr1^btk^* and *aip1.2-1* compared with *aip1.2-1;ctr1^btk^*. *n*=150 cells from 30 roots per genotype. Significance *P* values were determined by KS test. **P=0.000 *ctr1^btk^* versus *aip1.2-1*; *P*=0.882 *ctr1^btk^* versus *aip1.2-1;ctr1^btk^*; **P=0.000 *aip1.2-1* versus *aip1.2-1;ctr1^btk^*. (E) Quantitative analysis of trichoblast cell length in *aip1.2-1*, *ctr1^btk^* and *aip1.2-1;ctr1^btk^*. Average cell length from *n*=150 cells from 30 roots. Data are means±s.d. from three independent experiments, each employing 50 cells from ten roots. Statistical differences determined by two-tailed, unpaired *t*-test with equal variance and significance level *P*<0.05 are indicated as **P<0.01; *n*=3.

To further analyse the functional relationship between *AIP1-2* and *CTR1*, we generated an *aip1.2-1;ctr1^btk^* double mutant that exceptionally displayed basal-most placement of root hairs along trichoblasts and was indistinguishable from *ctr1^btk^* (Fig. 7A,C,D), but significantly differed from *aip1.2-1* seedlings (Fig. 7B-D). Moreover, average trichoblast length did not differ between *aip1.2-1;ctr1^btk^* and *ctr1^btk^* (Fig. 7E), although further reduction of trichoblast cell length is found in the strong *ctr1-1* allele (Ikeda et al., 2009). Together, these findings suggest that *CTR1* behaves epistatically over *AIP1-2* during trichoblast elongation and root hair positioning.

## DISCUSSION

In this study, we unravel a function for *ACT2* and *ACT7* downstream of *CTR1* during planar polarity formation (a schematic model is found in supplementary material Fig. S6). We further discover AIP1-2 as an interactor of ACTINs and as a negative modulator of planar polarity, the expression of which depends on WER and ethylene signalling. Although a role for *ACT2* during root hair positioning had been described (Ringli et al., 2002), our results reveal the strongest contribution of *ACT7* to planar polarity among the three actins tested. The phenotypes of *act2;act7* mutants further suggest synergistic interaction of the two genes during this process. Strikingly, analysis of double and triple mutants between *ctr1^btk^*, *act2-3* and *act7-6* placed the actin cytoskeleton downstream of ethylene signalling. Whereas ethylene signalling had been known to play a role in microtubule reorganisation (Steen and Chadwick, 1981; Lang et al., 1982; Roberts et al., 1985), our results reveal a genetic link with the actin cytoskeleton. Considering that upregulation of ethylene signalling promotes local auxin biosynthesis in the root tip (Ikeda et al., 2009), and that *ACT7* can be induced by auxin (Kandasamy et al., 2001), it is conceivable that some of the observed effects are mediated by auxin.

When searching for ACT7 interactors in a yeast two-hybrid screen, we identified AIP1-2, the yeast homologue Aip1p of which was discovered in a screen for interactors of Act1p (Amberg et al., 1995). Interestingly, neither yeast two-hybrid nor *in vitro* pull-down assays revealed an obvious difference between the interactions of AIP1-1 or AIP1-2 with the tested ACTs. Therefore, spatio-temporal differences in expression of the investigated proteins or the presence of other actin-binding proteins that have evolved class-specific interactions (Kandasamy et al., 2007) might confer specificity to ACT-AIP1 interaction *in vivo*.

Our analysis of *aip1-2* mutant alleles revealed a basal shift of root hair position, suggesting a negative role for *AIP1-2* during planar polarity formation. In contrast to decreased tissue polarisation in *act* mutants, which might result from destabilisation of actin filaments such as the one observed in *act2* single mutants (Kandasamy et al., 2009), the increase in tissue polarisation in *aip1-2* mutants could be explained by stabilisation of actin filaments into bundles, as observed in *Arabidopsis AIP1* RNAi lines (Ketelaar et al., 2004). However, we did not find an increased bundling of actin in *aip1-2* mutants. This is most likely due to the subtle phenotypic defects that might not allow resolving differences in actin organisation when compared with the actin bundling in root epidermal cells observed in RNAi lines downregulated for both *Arabidopsis AIP1-1* and *AIP1-2* (Ketelaar et al., 2004). Increased actin bundling has also been observed in knockout mutants of the single *AIP1* gene in the moss *Physcomitrella patens* (Augustine et al., 2011) and in hypomorphic *flr* mutant alleles in *Drosophila* (Ren et al., 2007). Interestingly, *flr* negatively affects F-actin accumulation and is required for establishment of wing hair polarity downstream of Frizzled signalling (Ren et al., 2007), suggesting evolutionary conservation of *AIP1*-mediated actin reorganisation during planar polarity formation in diverse organisms.

In contrast to single-celled yeast, the interaction between specific actin isoforms and AIP1-homologues has not been studied during development of multicellular organisms. To find out which actins might interact with AIP1-2 during planar polarity formation in roots, we studied double mutants between *aip1-2* and mutant alleles of three *ACTs*, revealing strong genetic interaction between *AIP1-2* and *ACT7*. Consistent with these findings, *ACT2* and *ACT8* function is mainly required during the later stage of root hair tip growth (Kandasamy et al., 2009). Accordingly, double mutants between *aip1-2* and *act2* or *act8* only showed weak defects in root hair positioning (C.S.K. and M.G., unpublished). Interestingly, the synthetic lethality of *act7;aip1-2* mutants resembles the situation in yeast, in which deletion of *AIP1* is synthetically lethal only in combination with a hypomorphic *act1* mutant allele (Rodal et al., 1999). Whereas analyses of single and double mutants suggest that AIP1-2 primarily interacts with ACT7 during planar polarity formation, the lethality of most *act7;aip1-2* seeds provides the possibility for future analyses of this interaction during earlier stages of development, including embryogenesis.

The early action of *AIP1-2* and *ACT7* during planar polarity formation is further supported by the mis-positioning of ROP patches in these mutants, demonstrating a requirement for actin during polar ROP placement. As short-term treatments with F-actin-destabilising drugs did not affect ROP localisation during hair initiation (Molendijk et al., 2001), it is conceivable that the actin cytoskeleton affects polar ROP recruitment in an indirect manner. Nevertheless, whereas previous studies highlighted a role for actin downstream of ROP signalling (Fu et al., 2005, 2009; Nagawa et al., 2012; Lin et al., 2012), our findings reveal an additional requirement of AIP1-2 and ACT7 upstream of ROPs in planar polarity formation. In addition, patterned expression of AIP1-2 in hair files from the early elongation zone onwards supports an early role of actin reorganisation during planar polarity formation. This cell type-enriched AIP1-2 expression depends on *WER* function as well as on balanced levels of the phytohormones ethylene and auxin. The strong ectopic expression of AIP1-2 observed in *wer-1* suggests that the WER pathway is at the core of specifying AIP1-2 expression, whereas the two hormones might modify events downstream of WER action, as previously suggested for their relationship towards other components of the cell fate machinery (Masucci and Schiefelbein, 1996). Consistent with this idea, auxin induces expression of *RSL4*, a downstream transcription factor of the WER pathway that positively regulates genes specifically expressed in hair cells with a function during hair differentiation (Yi et al., 2010). However, *AIP1-2* was not identified among the genes regulated by RSL4, suggesting the existence of additional components or mechanisms that act to integrate hormone- and WER-controlled signals. Intriguingly, our observations that WER mediates patterned expression of AIP1-2 by restricting its expression to hair cell files is contrasted by the findings that expression of the AUX1 auxin influx carrier, which also contributes to planar polarity (Grebe et al., 2002), requires the function of WER and its closest homologue MYB23 for expression in non-hair cell files (Jones et al., 2009). These findings support the view that WER function negatively affects expression of factors involved in planar polarity in hair and non-hair files. Interestingly, whereas components of the Frizzled planar polarity pathway in *Drosophila* are uniformly expressed throughout the tissue layer, similar to downstream effectors of cytoskeletal organisation, such as Flr and Tsr (Goodrich and Strutt, 2011), our work reveals AIP1-2 as a component of the planar polarity pathway in *Arabidopsis* that is under control of a patterning system spatially restricting its expression. Our work thus opens the door for future studies on molecular and cellular mechanisms executing planar polarity, such as cytoskeletal reorganisation and its control by a plant-specific patterning system.

## MATERIALS AND METHODS

### Plant material and growth conditions

The following mutant lines of *Arabidopsis thaliana* accession Columbia-0 (Col-0) were obtained from the European Nottingham Arabidopsis Stock Centre (NASC): *act2-3* (stock number N6959), *act7-6* (N670149), *act7-7* (N447790), *act8-2* (N446015), Col-8 (N60000; genetically equivalent to Col-0), *aip1.2-1* (N406016), *aip1.2-2* (N873278) and *wer-1* (N6349; Lee and Schiefelbein, 1999). Further mutant or marker lines were provided by the corresponding authors of the following articles: *ctr1^btk^* (Ikeda et al., 2009), *GFP-FABD;mCherry-TUA5* (Sampathkumar et al., 2011; Ketelaar et al., 2004; Gutierrez et al., 2009), *GFP-ABD2-GFP* (Wang et al., 2008), *Lifeact-Venus* (Era et al., 2009), *WAVE 1Y* (Geldner et al., 2009). Primers employed for genotypic characterisation of the lines mentioned above are listed in supplementary material Table S2.

*Arabidopsis* plants were grown under a photoperiod of 16 h light (150 µmol/m^2^/s) at 22°C and 8 h dark at 18°C at 60% air humidity. Seeds were surface-sterilised and stratified as described (Fischer et al., 2006). Seedlings were vertically grown for five days on Murashige and Skoog (MS) medium (1× MS salt; Duchefa, Haarlem, The Netherlands), 10 g/l sucrose, 8 g/l plant agar (Duchefa), 0.5 g/l 2-(N-morpholino)ethanesulfonic acid hydrate (MES; Sigma-Aldrich, pH 5.7). Media containing 5 µM ACC (Sigma-Aldrich), 20 nM 2,4-D (Sigma-Aldrich) or 100 nM 1-NAA (Sigma-Aldrich) were prepared as described (Grebe et al., 2002; Fischer et al., 2006).

An *Arabidopsis* root-cell suspension culture (Col-0; Boutté et al., 2010) was grown in MS medium for *Arabidopsis* [MSAR; 1× MS basal salts (Sigma-Aldrich), 30 g/l sucrose, 0.24 mg/l 2,4-D, 14 µg/l kinetin (Sigma-Aldrich), 200 mg/l *myo*-inositol (Sigma-Aldrich), 1.0 mg/l nicotinic acid (Sigma-Aldrich), 1.0 mg/l pyridoxine (Sigma-Aldrich), 10.0 mg/l thiamine (Sigma-Aldrich), pH 5.7] under constant shaking. The cell suspension was sub-cultured weekly by diluting it 6.7-fold in fresh medium.

### Quantitative analysis of root hair positioning

Quantitative analysis of relative root hair positions was performed as described previously (Fischer et al., 2006). One-hundred and fifty cells per genotype were analysed derived from three independent experiments performed on ten roots, 50 cells each. As root hair positioning does not follow a normal distribution, differences in distributions of relative hair position between genotypes were tested for significance using the non-parametric, two-sample Kolmogorov–Smirnov (KS)-test (http://www.physics.csbsju.edu/stats/KS-test.n.plot_form.html) as described (Pietra et al., 2013). The KS-test considers both location and shape of the distributions. Significance level was set at *P*<0.05.

### Immunocytochemistry and quantitative analysis of ROP positioning

Whole-mount immunolabelling of roots was performed as described (Boutté and Grebe, 2014), in three independent experiments with two different secondary antibodies. Antibody dilutions were: affinity-purified rabbit anti-AtROP2 1:250 (generation of antibody is described in the supplementary material); DyLight 488 donkey anti-rabbit (Jackson ImmunoResearch, 711-485-152; 1:400); DyLight 549 donkey anti-rabbit (Jackson ImmunoResearch, 711-505-152; 1:400). Nuclei were counterstained with DAPI (1 µg/ml, Sigma-Aldrich). Immunolabelled roots were imaged by confocal laser scanning microscopy (CLSM) using a Zeiss LSM780 system. Images were processed and analysed using ImageJ (NIH) and Adobe Photoshop CS4 (Adobe), and ROP positioning in trichoblasts prior to root hair initiation was quantified as previously described (Fischer et al., 2006). As relative position of ROP patches along trichoblasts did not follow a normal distribution, significances of differences between distributions were analysed employing the KS-test with a significance level at *P*<0.05 as described (Pietra et al., 2013).

### Bacterial protein expression and protein extraction

*AIP1-1* and *AIP1-2* cDNA sequences were cloned C-terminally of the GST-coding sequence, between the *Nco*I and the *Kpn*I restriction sites of the pET-GST1b vector. This is a derivative of pET-GST1a (Bogomolovas et al., 2009; kindly provided by Dr Gunter Stier, Heidelberg University, Germany), in which the 6×Histidine (6×His) sequence is introduced C-terminally of the GST moiety instead of N-terminally as in pET-GST1a. A GST-only control vector was generated by introducing a STOP linker into the *Nco*I restriction site. Oligonucleotide sequences used for cloning are listed in supplementary material Table S2. Vectors encoding GST or GST-fusion proteins were transformed into *E. coli* Rosetta DE3 cells (Merck). According to an auto-induction protocol for protein expression in bacteria (Studier, 2005), single Rosetta DE3 colonies containing the GST-fusion expression vectors were transferred to 3 ml auto-inducing ZYM 5052 medium [1% N-Z-amine (Sigma-Aldrich), 0.5% yeast extract, 25 mM Na_2_HPO_4_, 25 mM KH_2_PO_4_, 50 mM NH_4_Cl, 5 mM Na_2_SO_4_, 2 mM MgSO_4_, 0.5% glycerol, 0.05% glucose, 0.2% lactose] supplemented with trace elements (50 µM FeCl_3_, 20 µM CaCl_2_, 10 µM MnCl_2_, 10 µM ZnSO_4_, 2 µM CoCl_2_, 2 µM CuCl_2_, 2 µM NiCl_2_, 2 µM Na_2_MoO_4_, 2 µM H_3_BO_3_), initially incubated for 3 h at 37°C under constant shaking before the temperature was reduced to 20°C for another 16 h of growth. The pelleted cultures were resuspended in 300 µl lysis buffer [140 mM NaCl, 2.7 mM KCl, 10 mM Na_2_HPO_4_, 1.8 mM KH_2_PO_4_, 1× protease inhibitor cocktail (Sigma-Aldrich), 1 mM phenylmethanesulfonyl fluoride (PMSF; Sigma-Aldrich), pH 7.3] and lysed using a standard sonication protocol (Ausubel et al., 1995). Triton X-100 was added to a relative concentration of 1% and the protein extracts were collected after 10 min centrifugation at 15,000 ***g***.

6×His-fusions of ACT2 and ACT7 were expressed from the pColdI vector (Takara). *ACT2* and *ACT7* cDNAs were cloned C-terminally of the 6×His moiety between the *Bam*HI and the *Eco*RI restriction sites of the vector. Primers used for cloning are listed in supplementary material Table S2. According to a modified isopropyl β-D-thiogalactoside (IPTG) induction protocol (Tamura et al., 2011), single *E. coli* Rosetta DE3 colonies containing the 6×His-fusion expression vectors were transferred to 5 ml LB medium (10 g/l bacto-tryptone, 5 g/l bacto yeast extract, 5 g/l NaCl) and incubated for 4 h at 37°C under constant shaking. The cells were then cooled to 15°C for 30 min, IPTG (Sigma-Aldrich) was added to a final concentration of 50 µM and the cultures were incubated for 20 h at 15°C under constant shaking. According to the protocol of Tamura et al. (2011), the pelleted cultures were cooled down to −80°C for 2 h, resuspended in 1 ml buffer A [1 mM ethylenediamine tetraacetic acid (EDTA), 5 mM DL-dithiothreitol (DTT), 1 mM adenosine 5′-diphosphate (ADP), 0.1 mM PMSF, 2 mM diisopropyl fluorophosphate (DIFP), 10 mM tris(hydroxymethyl)aminomethane (Tris), pH 8.0], lysed using a standard sonication protocol (Ausubel et al., 1995) and protein extracts were collected after 10 min centrifugation at 15,000 ***g***.

### Extraction of plant proteins

Extraction of plant proteins from a Col-0 root-cell suspension culture (Boutté et al., 2010) was performed as described previously (Grebe et al., 2000). Four days after subculturing, a 50 ml suspension culture was filtered through grade-3 qualitative filter paper (Munktell Filter, Falun, Sweden). Cells were frozen in liquid nitrogen, homogenised and suspended in 6 ml bead-binding buffer (50 mM K_3_PO_4_, 150 mM KCl, 1 mM MgCl_2_, pH 7.5), containing 1× protease inhibitor cocktail for use with plant cell extracts (Sigma-Aldrich) and 1 mM PMSF. Cells were disrupted by sonication using a standard protocol as applied to bacterial protein extracts (Ausubel et al., 1995), and 6 ml bead-binding buffer, containing 10% glycerol, 2% Triton X-100 and 1 mM DTT, was added. After initial centrifugation for 10 min at 10,000 ***g***, the supernatant was transferred to a fresh vial, centrifuged for 10 min at 30,000 ***g*** and the supernatant containing plant proteins was used for western blotting and *in vitro* binding assays.

### *In vitro* binding assays

We prepared a bead volume of 40 µl glutathione sepharose (Amersham) according to the manufacturer's protocol and applied 300 µl bacterial extracts containing GST, GST-AIP1-1 or GST-AIP1-2. Beads pre-absorbed with GST constructs or beads just after preparation according to the manufacturer's protocol were incubated with 300 µl plant extract obtained from root-cell suspension culture or 300 µl bacterial extracts containing 6×His-ACT2 or 6×His-ACT7. Following steps of the *in vitro* binding assay were conducted as previously described (Grebe et al., 2000).

### Western blot analyses

Dilutions and distributors of antibodies employed for western blot detection are listed in supplementary material Table S3. Enhanced chemiluminescence (ECL) prime western blotting detection reagents were obtained from Amersham and used according to the manufacturer's instruction. AGFA Cronex 5 medical X-ray films (Agfa-Gevaert) were developed using a Curix 60 processor (Agfa-Gevaert) or images of the blots were acquired using a LAS-3000 imaging system (Fujifilm).

### Yeast two-hybrid analyses

Initial screening for interactors of the full-length *Arabidopsis* ACT7 bait was performed at Hybrigenics (Paris, France) using ULTImate Y2H technology to screen a one-week-old *Arabidopsis* seedling cDNA expression library. Several full-length AIP1-2 (At3g18060) cDNA clones revealed it as the only interactor. Different *ACTIN* and *AIP1* cDNA sequences from *Arabidopsis* were re-cloned in original plasmid vectors of the LexA-based interaction trap version of the two-hybrid system (Gyuris et al., 1993; Ausubel et al., 1995), employing the pJG4-5 derivative pMG5 and the pEG202 derivative pMG8 (Grebe et al., 2000) as prey and bait vectors, respectively. Coding sequences were cloned in frame between the *Not*I and *Xba*I restriction sites, except for *ACT1* cDNA, which was cloned solely employing the *Not*I restriction site. Primers used for cloning of the analysed constructs and accession numbers for cDNA sequences are listed in supplementary material Table S2.

### Reverse transcription (RT)-PCR

Isolation and purification of mRNA from roots of five-day-old *Arabidopsis* seedlings and subsequent cDNA synthesis were performed as described (Pietra et al., 2013). For semi-quantitative PCR, primer pairs were employed that flanked the T-DNA insertions in *AIP1-2* and, furthermore, spanned introns in order to detect potential genomic DNA contaminations (see supplementary material Table S2 for primer sequences). PCR products were analysed after 28 cycles.

### Complementation tests

The genomic *AIP1-2* sequence (*gAIP1-2*) was cloned into the pGreenII0229 vector (John Innes Centre, Norwich, UK; Hellens et al., 2000) between the *Xba*I and *Apa*I restriction sites. The 2162 bp region upstream of the start codon of *AIP1-2* was employed as a promoter and cloned between the *Not*I and the *Xba*I restriction sites. The 465 bp region downstream of the stop codon of *AIP1-2* was employed as terminator and cloned into the *Kpn*I restriction site. The *3×Myc* coding sequence provided in supplementary material Table S2 was synthesised by GenScript, based on the *3×Myc* sequence from pNIGEL 07 (Geldner et al., 2009) and provided in the vector pUC57. The coding sequence for mCherry obtained in the plasmid pmCherry (Clontech) was amplified by PCR, and 3×Myc was restricted from pUC57-3×Myc using *Apa*I and *Kpn*I. It was then fused in-frame to the 3′-end of the *gAIP1-2* coding sequence using the *Apa*I and the *Kpn*I restriction sites. Primers used for cloning are listed in supplementary material Table S2. Integrity of all constructs was confirmed by nucleic acid sequencing. *Arabidopsis* plants ecotype Col-0 were transformed by the floral dip method (Clough and Bent, 1998) employing the *Agrobacterium tumefaciens* strain GV3101 (Koncz and Schell, 1986).

### Confocal laser scanning microscopy (CLSM)

All analyses using CLSM were conducted with a Zeiss LSM780 laser scanning microscope (Zeiss). Samples mounted in aqueous solutions were imaged using a water-corrected C-Apochromat 40× objective (Zeiss) with a numerical aperture (NA) of 1.2. Samples mounted in glycerol-based solutions were imaged using an oil-corrected Plan-Apochromat 63× objective with NA 1.4 (Zeiss). Detailed CLSM settings are listed in supplementary material Table S4.

### Visualisation of F-actin networks and AIP1-2-mCherry expression

F-actin networks at root hair initiation sites were visualised in seedlings expressing *GFP-FABD*, *GFP-ABD2-GFP*, *Lifeact-Venus* or in seedlings probed with BODIPY FL phallacidin (Nishimura et al., 2003). For display, images of early trichoblast cells acquired on longitudinal cross-sections at distances of 0.52 µm (GFP-FABD or Lifeact-Venus), 0.91 µm (GFP-ABD2-GFP) or 0.45 µm (BODIPY FL phallacidin) from each other, respectively, were processed by maximum intensity projection, employing ZEN lite 2012 analysis software (Zeiss).

To visualise cell file-specific AIP1-2-mCherry expression, longitudinal sections at a distance of 3.54 µm from a top view of the analysed seedling root were processed by maximum-intensity projection, employing ZEN lite 2012 analysis software. Cross-sectional overviews were generated by transversally projecting selected areas of 15.1 µm in length into one image.

### Image processing

Image analysis software programs used for acquiring and processing graphical data were: AxioVs40 V 4.8.2.0 (Zeiss), ZEN lite 2012 analysis software, ImageJ (NIH), Adobe Photoshop CS3 and CS4 and Adobe Illustrator CS4 (Adobe).

## Supplementary Material

Supplementary Material
