# Supplementary material for: Arabidopsis AIP1-2 restricted by WER-mediated patterning modulates planar polarity
Source: Development. 2015 Jan 1;142(1):151–61. doi: 10.1242/dev.111013 (PMC4299142; doi:10.1242/dev.111013)
Supplement: Supplementary Material [file supp_142_1_151__index.html]

Supplementary Material 

# *Arabidopsis AIP1-2* restricted by *WER*-mediated patterning modulates planar polarity

## DEV111013 Supplementary Material

**Files in this Data Supplement:**

- Supplementary Material
