# Supplementary material for: Arabidopsis AIP1-2 restricted by WER-mediated patterning modulates planar polarity
Source: Development. 2015 Jan 1;142(1):151–61. doi: 10.1242/dev.111013 (PMC4299142; doi:10.1242/dev.111013)
Supplement: Supplementary Material [file supp_dev.111013_DEV111013supp.pdf]

SUPPLEMENTARY MATERIALS AND METHODS

Analysis of actin filament organisation

To quantitatively evaluate actin cytoskeleton organisation we measured skewness of F-actin fluorescence in trichoblasts of five-day-old seedlings probed with Bodipy FL phallacidin as described previously (Higaki et al., 2010; van der Honing et al., 2012). Images were processed using ImageJ. Individual trichoblasts were isolated by manually excising the cells in each z-layer of stacks covering the full cells. Maximum intensity projections of isolated trichoblasts were processed with the rolling ball background subtraction (rolling ball radius set to 20 pixels) and the Gaussian blurring tool (sigma radius set to 1 pixel). The preprocessed images were skeletonised and the skewness of the actin cytoskeleton measured according to Higaki et al., 2010. Significance of differences between distributions was tested using the non-parametric, two-sample Kolmogorov-Smirnov (K-S)-test (http://www.physics.csbsju.edu/stats/KS-test.n.plot_form.html) with the significance level set at $P < 0.05$.

To evaluate the relative thickness of actin bundles, individual trichoblasts were isolated by manually excising the cells in each z-layer of stacks covering the full cells. Maximum intensity projections of isolated trichoblasts were processed with the rolling ball background subtraction tool and a rolling ball radius set to 20 pixels. Fluorescence intensity profiles of lines drawn perpendicular to the longitudinal axis of trichoblasts were obtained using ImageJ. Lines were drawn at relative distances of 1/10 and 2/10 of the full cell length from apical and basal ends of cells. Fluorescent maxima that were 10 arbitrary units higher than the surrounding were counted and the frequency of fluorescent peaks determined in classes ranging from 0-40, 40-80, 80-120 and 120< as described previously (van der Honing et al.,

2012). Significance of differences between distributions was tested using the χ^2 test with the significance level set at $P < 0.05$.

Protein extraction from seedlings

Five-day-old seedlings were frozen in liquid nitrogen and ground with a fine pestle in 200 µl extraction buffer (25 mM Tris-HCl pH 7.5, 10 mM $MgCl_2$, 5 mM EGTA, 10% glycerol, 100 mM NaCl, 0,2% Tween-20, 10 µl/ml Proteinase inhibitor cocktail (Sigma Aldrich), 2 mM DTT per 20 seedlings. Supernatants were collected after a 10 minutes centrifugation at 11,000 g and used for Western blot analyses.

Generation of rabbit anti-AtROP antibodies

Polyclonal anti-AtROP antibodies were produced in two rabbits (Calvin and Vickie) injected with a synthetic peptide corresponding to AtROP2 amino acids 124–138 (C-DDKQFFIDHPGAVPI) which had been N-terminally coupled to keyhole limpet hemocyanin via the thiol residues of an additional cysteine. anti-AtROP2 antibodies were affinity purified against the column-coupled DDKQFFIDHPGAVPI peptide (Agrisera, Vännäs, Sweden). Using a Mini-PROTEAN II Multiscreen apparatus (BioRad, Hercules, California), serial dilutions of anti-AtROP2 derived from rabbit Vickie purified at pH 7 in TBST (250 mM Tris/HCl, 750 mM NaCl, 0.05% Tween-20, pH 8.0) supplemented with 2% milk powder (Semper AB, Sundbyberg, Sweden) were tested to establish the optimal working concentration for western blot detection of endogenous ROPs (supplementary material Fig. S5A). At an optimal dilution of 1:500, the antibody specifically recognised proteins with the

size of ROP and GFP-ROP6 or EYFP-ROP2 in protein extracts from five-day-old Col, *p35S:GFP-ROP6* (Fu et al., 2009), *pROP6:GFP-ROP6* (Fu et al., 2009) or *pROP2:EYFP-ROP2* (Xu and Scheres, 2005) seedlings (supplementary material Fig. S5A,B). Equal loading of the gel and membrane were assessed by Coomassie Brilliant blue Staining and Western blot analysis using mouse monoclonal anti-plant ER BiP antibody (1:1000, Nordic Biosite AB, Täby, Sweden) and goat anti-mouse IgG-HRP (1:3.000, Biorad, Hercules, California, USA; 172-1011) (supplementary material Fig. S5B).

In whole-mount immunofluorescences anti-AtROP2 from rabbit Calvin purified at pH 2.5 was used in a dilution of 1:250 (in 50% glycerol) followed by DyLight633 conjugated donkey anti-rabbit IgG antibody (1:100, Agrisera, Vännäs, Sweden; AS12 2034) (supplementary material Fig. S5C). The ability to recognise ROP2 was determined by co-localising the anti-AtROP2 derived signal with EYFP-ROP2 in the root meristem and in root hair bulges of five-day-old transgenic *pROP2:EYFP-ROP2* seedlings (supplementary material Fig. S5C,D). Detailed CLSM settings are listed in supplementary material Tab. S3.

SUPPLEMENTARY REFERENCES

Fu, Y., Xu, T., Zhu, L., Wen, M. and Yang, Z. (2009). A ROP GTPase signaling pathway controls cortical microtubule ordering and cell expansion in *Arabidopsis*. *Curr. Biol.* **19**, 1827-1832.

Higaki, T., Kutsuna, N., Sano, T., Kondo, N. and Hasezawa, S. (2010). Quantification and cluster analysis of actin cytoskeletal structures in plant cells: role of actin bundling in stomatal movement during diurnal cycles in Arabidopsis guard cells. *Plant J.* **61**, 156-165.

van der Honing, H. S., Kieft, H., Emons, A. M. and Ketelaar, T. (2012). Arabidopsis VILLIN2 and VILLIN3 are required for the generation of thick actin filament bundles and for directional organ growth. *Plant Physiol.* **158**, 1426-1438.

Xu, J. and Scheres, B. (2005). Dissection of Arabidopsis ADP-RIBOSYLATION FACTOR 1 function in epidermal cell polarity. *Plant Cell* **17**, 525-536.

SUPPLEMENTARY TABLES

Table S1.

Significance levels for differences in ectopic AIP1-2-mCherry expression, determined by Fisher's Exact test.

**Genotype / growth medium**	**Genotype / growth medium**	***P*-Value**
wt (*aip1.2-1;gAIP1-2-mCherry*) / MS	*wer-1* (*wer-1;aip1.2-1;gAIP1-2-mCherry*) / MS	0.00000
wt (*aip1.2-1;gAIP1-2-mCherry*) / MS	$ctr1^{btk}$ ($ctr1^{btk}$*;aip1.2-1;gAIP1-2-mCherry*) / MS	0.00683
wt (*aip1.2-1;gAIP1-2-mCherry*) / MS	wt (*aip1.2-1;gAIP1-2-mCherry*) / MS containing 5 µM ACC	0.00005
wt (*aip1.2-1;gAIP1-2-mCherry*) / MS containing 0.00014% EtOH	wt (*aip1.2-1;gAIP1-2-mCherry*) / MS containing 0.00014% EtOH + 20 nM 2,4-D	0.00024
wt (*aip1.2-1;gAIP1-2-mCherry*) / MS containing 1 µM NaOH	wt (*aip1.2-1;gAIP1-2-mCherry*) / MS containing 1 µM NaOH + 100 nM 1-NAA	0.03389

Table S2. Oligonucleotide list.

Application	Target	Name	Sequence (5' – 3')
Genotyping PCR	*act7-6* (SALK_131610)	ACT7F0587	CTG CTT CTC GAA TCT TCT GTA TCA TC
		ACT7R114	AAA TCA TGA TCA GTA GTC TTA CAC AT
	act7-7 (GK-498G06)	ACT7F538	CGT ACA ACC GGT TAG TTC TTA ACT C
		ACT7R1220	GTG CTG AGG GAT GCA AGG
	act8-2 (GK-480C07)	ACT8F0443	CTT GTG TTG GAT CTC GAT TAC G
		ACT8R696	AGC ATG TGG AAG TGA GAA ACC
	aip1.2-1 (GK-063F04)	AIP2F2871	CAG TGT CTG AAT TTG GTG TTT AGG
		AIP2R3638	GTG TTC CAG ATT CAT GGA GAG ATT C
	aip1.2-2 (SAIL_337_E05)	AIP2F3622	CCA TGA ATC TGG AAC ACA AAC ACT G
		AIP2R4199	GAG AGA AAG CAC AAT CTC AAG TCC C
	T-DNA primer	SAIL-LB2	GCT TCC TAT TAT ATC TTC CCA AAT TAC CAA TAC A
		SALK-LBa1	TGG TTC ACG TAG TGG GCC ATC G
		GK-o8760	GGG CTA CAC TGA ATT GGT AGC TC
RT-PCR	*aip1.2-1* (GK-063F04)	AIP1-2-GK-spanF	AAC ACC TGA TGG AAC CGA AG
		AIP1-2-GK-spanR	CCA TAG TGC TGT TTG GAG ACC
	aip1.2-2 (SAIL_337_E05)	AIP1-2-SAIL-spanF	GCT GAT TTG AAC AGA GAA GCA G
		AIP1-2-SAIL-spanR	ACG AAG CAG GTT TGT CCA CT
	APT1 (At1g27450)	APT1_F	GTT GAA TGT GCT TGC G
		APT1_R	CTT TAG CCC CTG TTG G
Cloning of constructs for yeast two-hybrid experiments	*ACT1* (At2g37620)	ACT1FnotI	GTG CGG CCG CAT GGC TGA TGG TGA AGA CAT TC
		ACT1RnotI	CAA GCG GCC GCT CAG AAG CAC TTC CTG TGA AC
	ACT2 (At3g18780)	ACT2FnotI	GCG GCC GCA TGG CTG AGG CTG ATG ATA TTC AAC C
		ACT2RsalI	GTC GAC TTA GAA ACA TTT TCT GTG AAC GAT TCC
	ACT7 (At5g09810)	ACT7FnotI	GCG GCC GCA TGG CCG ATG GTG AGG ATA TTC AGC
		ACT7RsalI	GTC GAC TTA GAA GCA TTT CCT GTG AAC AAT CG
	ACT8 (At1g49240)	ACT8NotI	GAT GCG GCC GCA TGG CCG ATG CTG ATG AC
		ACT8RxhoI	CTC CTC GAG TTA GAA GCA TTT TCT GTG GAC AAT G
	ΔAIP1-1 / AIP1-1 (At2g01330)	ΔAIP1FnotI	GGC GGC CGC ATG GGG GAT TTT GAT GGC
		AIP1FnotI	CCT ACC AAC AGC GGC CGC ATG GCG AAA CTC CTC GAG AC
		AIP1RsalI	AAC GTC GAC TCA CTG AGG TTC GAT ATG CC
	AIP1-2 (At3g18060)	AIP2F1NotI	GAT GCG GCC GCA TGG AGC TTT CAG AAA CCT ATG C
		AIP2RsalI	CGT GTC GAC TTA TTG AGG AGT AAA GCT CCA GAC G
Cloning of constructs for in vitro pull-down experiments	*AIP1-1* (At2g01330)	AIP1FncoI	GCT TCC ATG GCG AAA CTC CTC GAG AC
		AIP1RkpnI	GCT TGG TAC CTC ACT GAG GTT CGA TAT GCC A
	AIP1-2 (At3g18060)	AIP2FncoI	GCT TCC ATG GAG CTT TCA GAA ACC TAT GC
		AIP2RkpnI	GCT TGG TAC CTT ATT GAG GAG TAA AGC

			TCC AGA CG
	STOP linker	STOP-linkerF	CAT GGT GAC TGA CTG AG
		STOP-linkerR	GTA CCT CAG TCA GTC AC
	ACT2 (At3g18780)	ACT2FbamHI	GTC CGG ATC CGC TGA GGC TGA TGA TAT TCA AC
		ACT2RecoRI	GCT TGA ATT CTT AGA AAC ATT TTC TGT GAA CGA TTC
	ACT7 (At5g09810)	ACT7FbamHI	GTC CGG ATC CGC CGA TGG TGA GGA TAT TCA
		ACT7RecoRI	GCT TGA ATT CTT AGA AGC ATT TCC TGT GAA CAA TC
Cloning of a genomic rescue construct	*pAIP1-2* (At3g18060)	pAIP2FnotI	TAA GCG GCC GCT ACC CTG AAG CGT TGT TGG
		pAIP2RXbaI	AAA GTC TAG ACA GAA CCG AGG ACT TTG CAG
	AIP1-2 (At3g18060)	AIP2FXbaITG	CCT TCT AGA TGA TGG AGC TTT CAG AAA CC
		AIP2R1827ApaI	CAA GGG CCC TTG AGG AGT AAA GCT CCA GAC G
	mCherry	mChF1ApaI	GGG CCC ATG GTG AGC AAG GGC GAG
		mChRStKpnI	GGT ACC TTA CTT GTA CAG CTC GTC CAT GC
	3xMyc	3xMyc	GAG CAA AAG CTC ATT TCT GAA GAG GAC TTG AAT GGA GAA CAG AAA TTG ATC AGT GAG GAA GAC CTC AAC GGT GAG CAG AAG TTA ATA TCC GAG GAG GAT CTT AAT TAA
	3'utrAIP1-2	3UAIP2FKpnI	GTC TGG TAC CTT ACT CCT CAA TAA GAC TTG ACG
		3UAIP2RBsrGI	GAG TGT ACA TGT GTG TCT TCT TGC TTC AGG

Table S3.

List of antibodies and the dilutions employed for Western blot detection.

**Antibody**	**Epitope**	**Dilution**	**Company**	**Order number**
rabbit anti-LexA	LexA	1:50,000	Abcam, Cambridge, UK	ab50953
rabbit anti-AtROP2	ROPs	1:500	---	---
donkey anti-rabbit IgG-HRP	rabbit anti-LexA<br>rabbit anti-AtROP2	1:100,000	Jackson ImmunoResearch, West Grove, Pennsylvania, USA	711-035-152
mouse anti-HA11	HA	1:1,000	Covance, Princeton, New Jersey, USA	MMS-101R
mouse anti-c-Myc	3xMyc	1:1,000	Santa Cruz Biotechnology, Dallas, Texas, USA	sc-40
mouse anti-ACT11	ACTINs	1:1,000	Agrisera, Vännäs, Sweden	AS10702
goat anti mouse IgG-HRP	mouse anti-HA11<br>mouse anti-c-Myc<br>mouse anti-ACT11	1:3,000	Biorad, Hercules, California, USA	172-1011
anti-GST-HRP	GST	1:10,000	GE Healthcare Bio-Sciences AB, Uppsala, Sweden	RPN1236

Table S4.

Confocal laser scanning microscopy settings for the analysed fluorophores.

Fluorophore/Fluorescent fusion	Excitation lines [nm]	Emission filter [nm]	Acquisition mode
GFP-FABD, GFP-ABD2-GFP, Bodipy FL phallacidin	488	496–598	Single
Lifeact-Venus	514	522-615	Single
Bodipy FL phallacidin + AIP1-2-mCherry	488, 561	493-552, 587-696	Single
WAVE 1Y + AIP1-2-mCherry	514, 561	522-573, 602-696	Single
AIP1-2-mCherry	561	578-696	Single
DAPI + DyLight488	405, 488	410-522, 530-683	Sequential line
DAPI + DyLight549	405, 561	410-564, 564-683	Sequential line
DAPI + DyLight633	405, 633	410-498, 638-759	Sequential line
EYFP-ROP2 + DyLight633 (anti-rabbit)	514, 633	522--573, 638-759	Single

SUPPLEMENTARY FIGURE LEGENDS

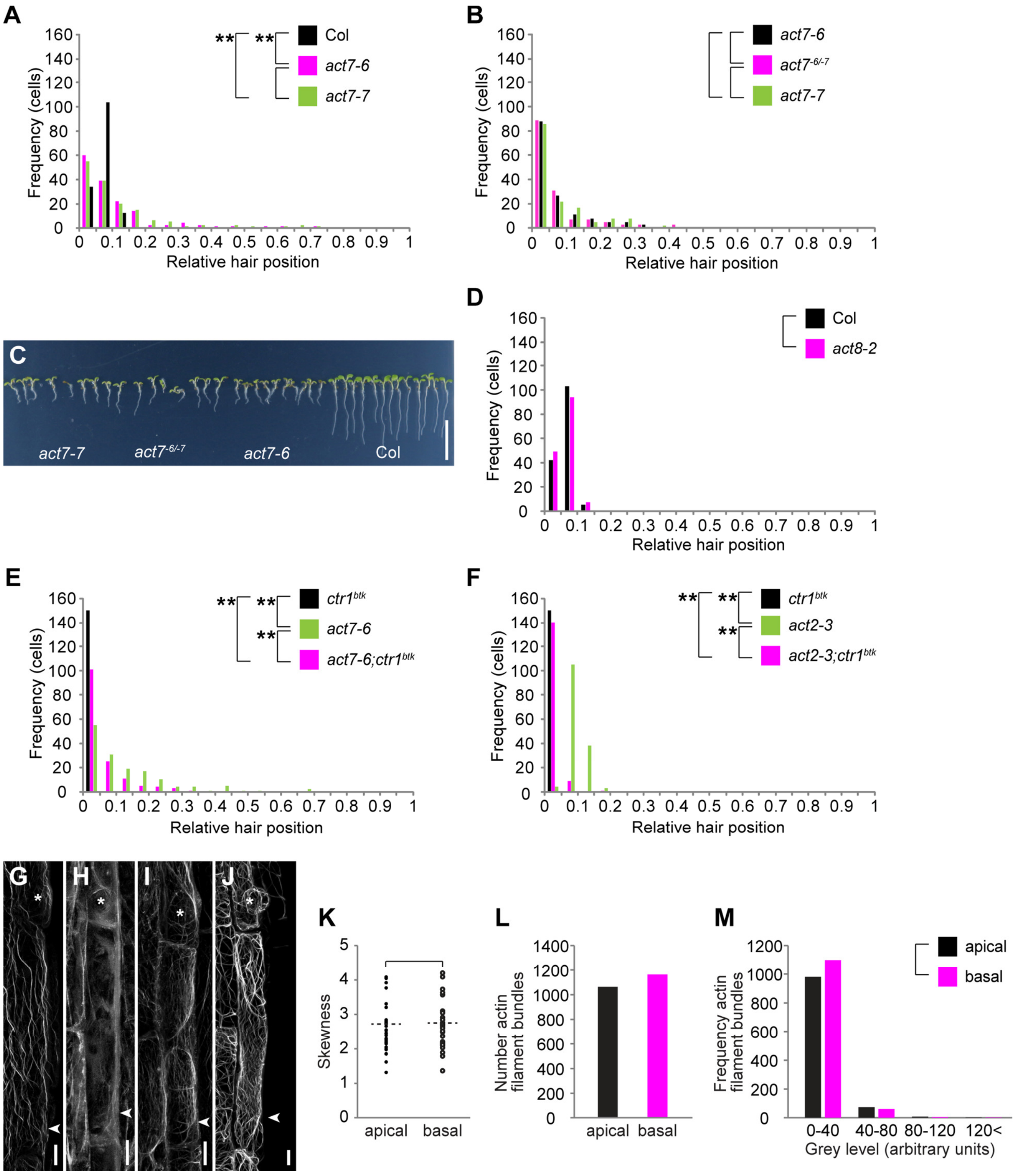

Fig. S1. *ACT2* and *ACT7* are required for planar polarity formation downstream of *CTR1* (additional data). (A,B) Quantitative analysis of relative root hair positioning phenotypes of *act7-6* and *act7-7* compared with (A) Col and (B) *act7-6*/*act7-7* (*act7*$^{-6/-7}$) transheterozygotes. n = 150 cells from 30 roots per genotype. Significances *P* were determined by the K-S test. (A) ***P* = 0.000 Col vs. *act7-6*; ***P* = 0.000 Col vs. *act7-7*; *P* = 0.607 *act7-6* vs. *act7-7*. (B) *P* = 0.607 *act7-6* vs. *act7-7*; *P* = 0.800 *act7-6* vs. *act7*$^{-6/-7}$; *P* = 0.422 *act7-7* vs. *act7*$^{-6/-7}$. (C) Five-day-old *act7-7*, *act7*$^{-6/-7}$, *act7-6* and Col seedlings. (D-F) Quantitative analysis of relative root hair positioning phenotypes of (D) *act8-2* compared with Col, (E) *ctr1*^{btk}, *act7-6* and *act7-6;ctr1*^{btk}, and (F) *ctr1*^{btk}, *act2-3* and *act2-3;ctr1*^{btk}. n = 150 cells from 30 roots per genotype. Significances *P* were determined by the K-S test. (D) *P* = 0.882 Col vs. *act8-2*. (E) ***P* = 0.000 *ctr1*^{btk} vs. *act7-6*; ***P* = 0.000 *ctr1*^{btk} vs. *act7-6;ctr1*^{btk} and ***P* = 0.000 *act7-6* vs. *act7-6;ctr1*^{btk}. (F) ***P* = 0.000 *ctr1*^{btk} vs. *act2-3*; ***P* = 0.000 *ctr1*^{btk} vs. *act2-3;ctr1*^{btk} and ***P* = 0.000 *act2-3* vs. *act2-3;ctr1*^{btk}. (G-J) CLSM projections of actin filaments (G) stained with Bodipy FL phallacidin or labelled with (H) GFP-FABD, (I) GFP-ABD-GFP or (J) Lifeact-Venus in root hair cells prior to hair outgrowth. (K) Skewness analysis of actin cytoskeleton organisation in the apical (apical) and basal (basal) fifth of 35 Col cells. The skewness value for each measured trichoblast is shown. Significance *P* was determined by K-S test. *P* = 0.64. (L) Number of actin filament bundles detected in the apical (apical) and basal (basal) fifth of 35 Col cells. (M) Frequency of actin filament bundles in apical (apical) and basal (basal) ends of Col cells, classified by signal intensities indicated on the x-axis. Significance *P* was determined by χ^2 test. *P* = 0.2625. Scale bars: 10 mm in C; 10 µm in G-J.

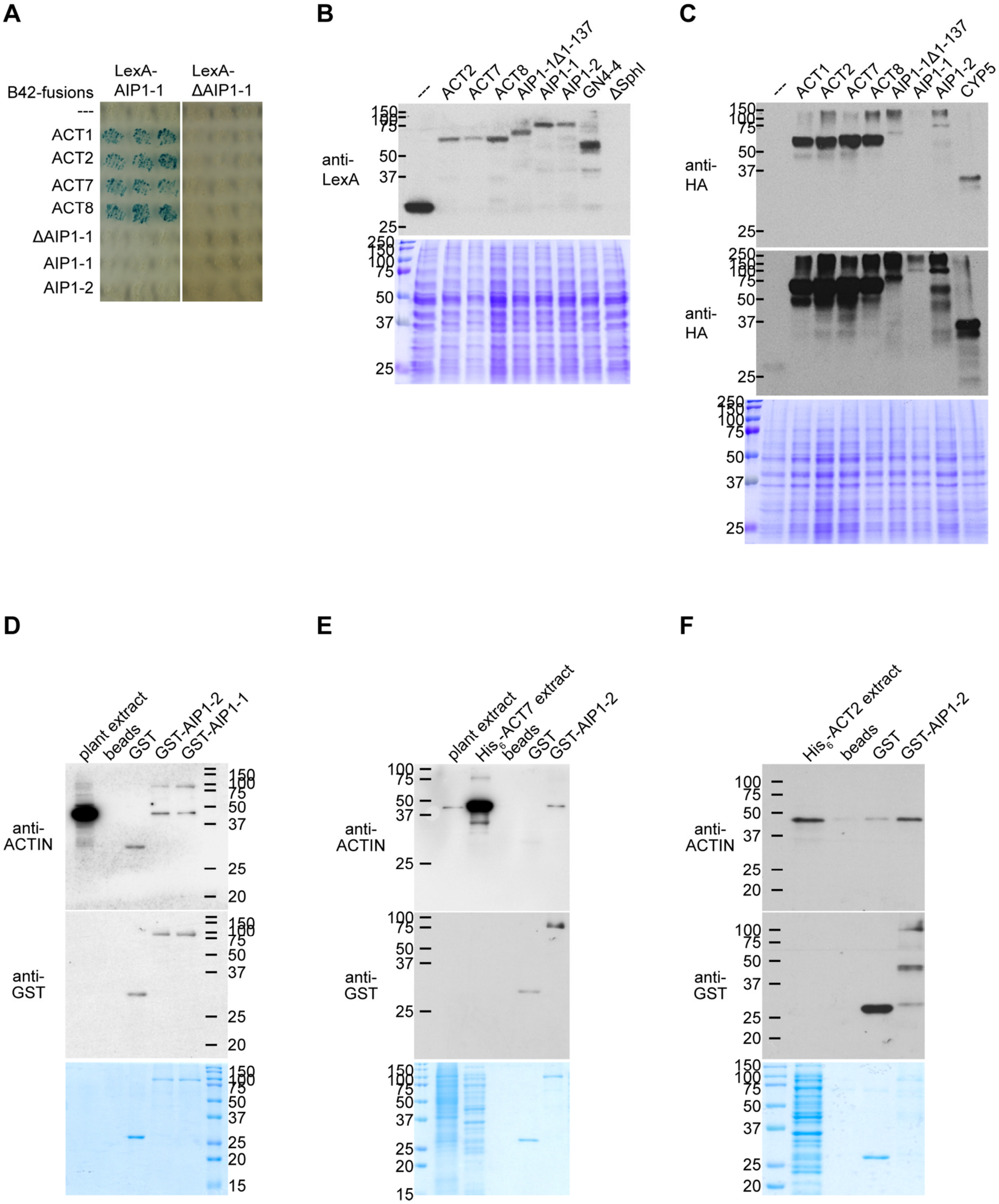
A
B
C
B42-fusions
LexA-AIP1-1
LexA-ΔAIP1-1

ACT1
ACT2
ACT7
ACT8
ΔAIP1-1
AIP1-1
AIP1-2
ACT2
ACT7
ACT8
AIP1-1Δ1-137
AIP1-1
AIP1-2
GN4-4
ΔSphI
anti-LexA
ACT1
CYP5
anti-HA
D
E
F
plant extract
beads
GST
GST-AIP1-2
GST-AIP1-1
anti-ACTIN
anti-GST
His6-ACT7 extract
His6-ACT2 extract

Fig. S2. AIP1-1 and AIP1-2 interact with ACTINs in yeast and *in vitro* (full blots and additional data). (A) LexA-AIP1-1 but not a LexA-□AIP1-1, which contains a truncated form of AIP1-1 lacking the first 137 amino acids, interacts with B42 transcriptional activation domain (AD)-fusions of ACT1, ACT2, ACT7 and ACT8. Yeasts were grown for 24 h (left panel) and 48 h (right panel) prior to imaging. Empty prey vector (---) and □AIP1-1 were used as negative controls. (B,C) Expression analysis of (B) LexA (bait) and (C) AD (prey) fusions in yeast clones used for yeast two-hybrid analyses. Lower panels are Coomassie Blue-stained gels with loading corresponding to Western blots in upper panels. (C) Upper two panels display anti-HA western blot with different exposure times. (D-F) Full blots and corresponding Coomassie Blue-stained gels with loading corresponding to Western blots displayed in Fig. 2.

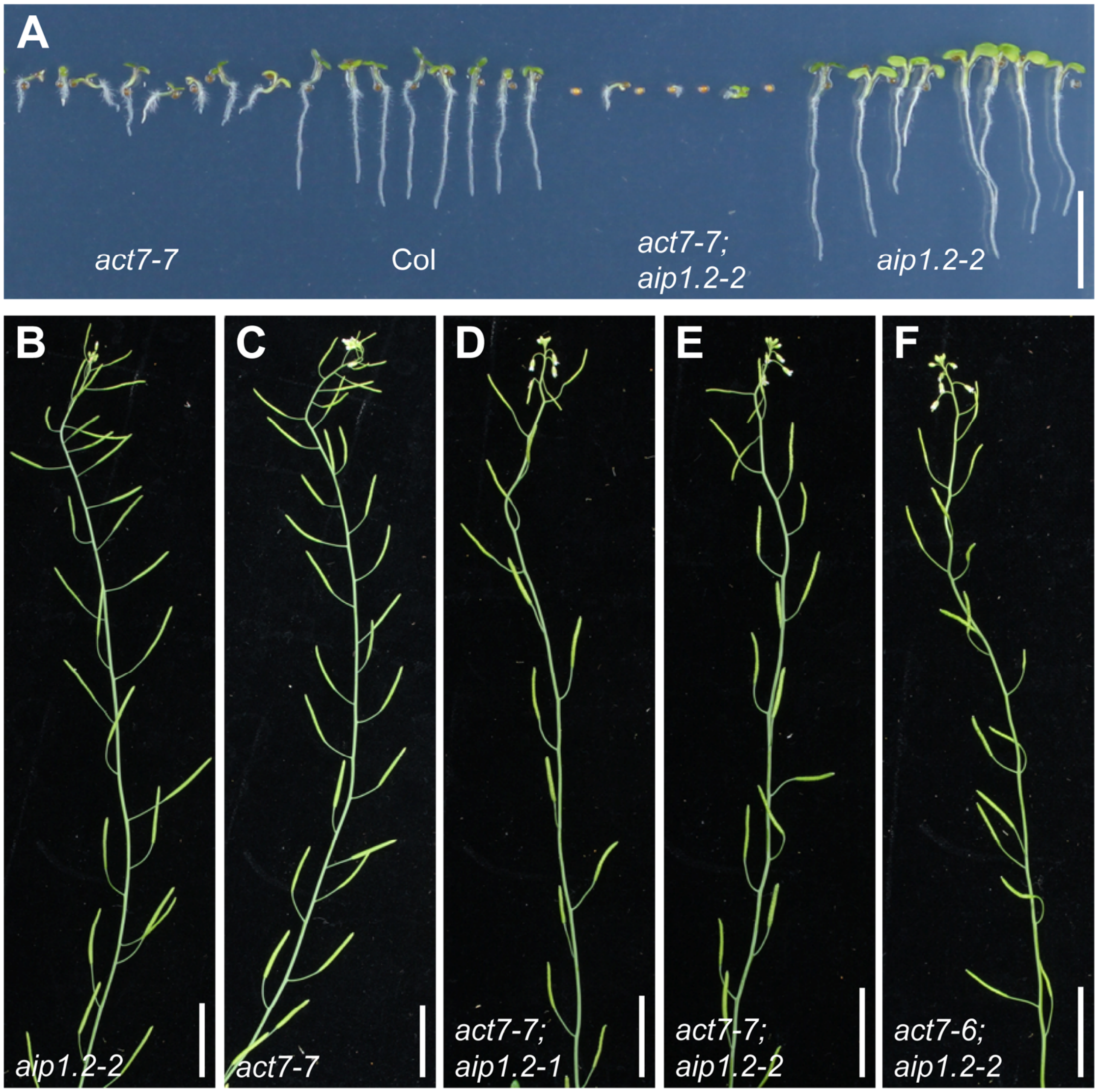

Fig. S3. *AIP1-2* and *ACT7* genetically interact in *Arabidopsis* (additional allele combinations). (A) Five-day-old *act7-7*, Col, *act7-7*, *act7-7;aip1.2-2* and *aip1.2-2* seedlings. Germination rate of *act7-7;aip1.2-2* seeds is strongly reduced compared to Col, *act7-7* and *aip1.2-2*. (B-F) Main stems of three-week-old *aip1.2-2*, *act7-7*, *act7-7;aip1.2-1*, *act7-7;aip1.2-2* and *act7-6;aip1.2-2* plants. Note, double mutants display defects in silique arrangement and orientation as well as shoot twisting that are absent in the single mutants. Scale bars: 10 mm in A; 20 mm in B-F.

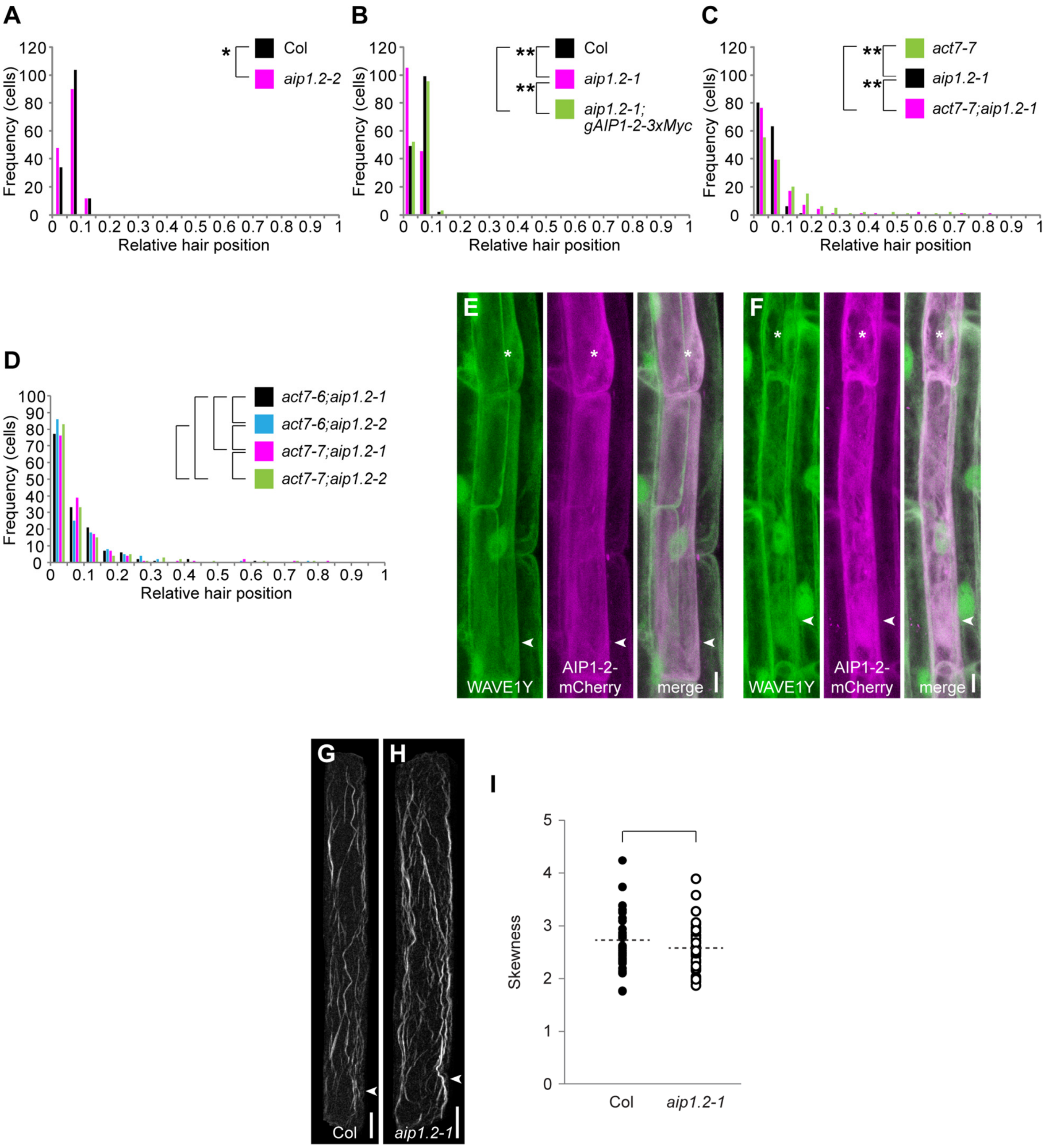

Fig. S4. ***AIP1-2*** **modulates planar polarity formation (additional alleles, rescue constructs and double mutants) and localises to the cytosol.** (A-C) Quantitative analysis of relative root hair positioning phenotypes of (A) *aip1.2-2* compared with Col, (B) *aip1.2-1* and *aip1.2-1;gAIP1-2-3xMyc* compared with Col and (C) *act7-7* and *aip1.2-1* compared with *act7-7;aip1.2-1*. n = 150 cells from 30 roots for all genotypes. Significances *P* were determined by K-S test. (A) **P* = 0.019 Col vs. *aip1.2-2*. (B) ***P* = 0.000 Col vs. *aip1.2-1; P*

= 0.800 Col vs. *aip1.2-1;gAIP1-2-3xMyc* and ***P* = 0.000 *aip1.2-1* vs. *aip1.2-1;gAIP1-2-3xMyc*. (C) ***P* = 0.000 *act7-7* vs. *aip1.2-1*; *P* = 0.053 *act7-7* vs. *act7-7;aip1.2-1* and ***P* = 0.000 *aip1.2-1* vs. *act7-7;aip1.2-1*. (D,E) CLSM projection images of *WAVE 1Y*$^{+/-}$*;aip1.2-1*$^{+/-}$*;gAIP1-2-mCherry*$^{+/-}$ seedlings from a (D) longitudinal and (E) tangential cross section of a root. Asterisks mark bulge sites and arrowheads future hair initiation sites. (F,G) CLSM projection images of isolated (F) Col and (G) *aip1.2-1* trichoblasts prior to hair bulging probed with Bodipy FL phallacidin. Arrowheads indicate future hair initiation sites. (H) Skewness analysis of actin cytoskeleton organisation. The skewness value for each measured trichoblast is shown. n = 35 for Col and n = 32 for *aip1.2-1*. Significance *P* was determined by K-S test. *P* = 0,642. Scale bars: 10 µm.

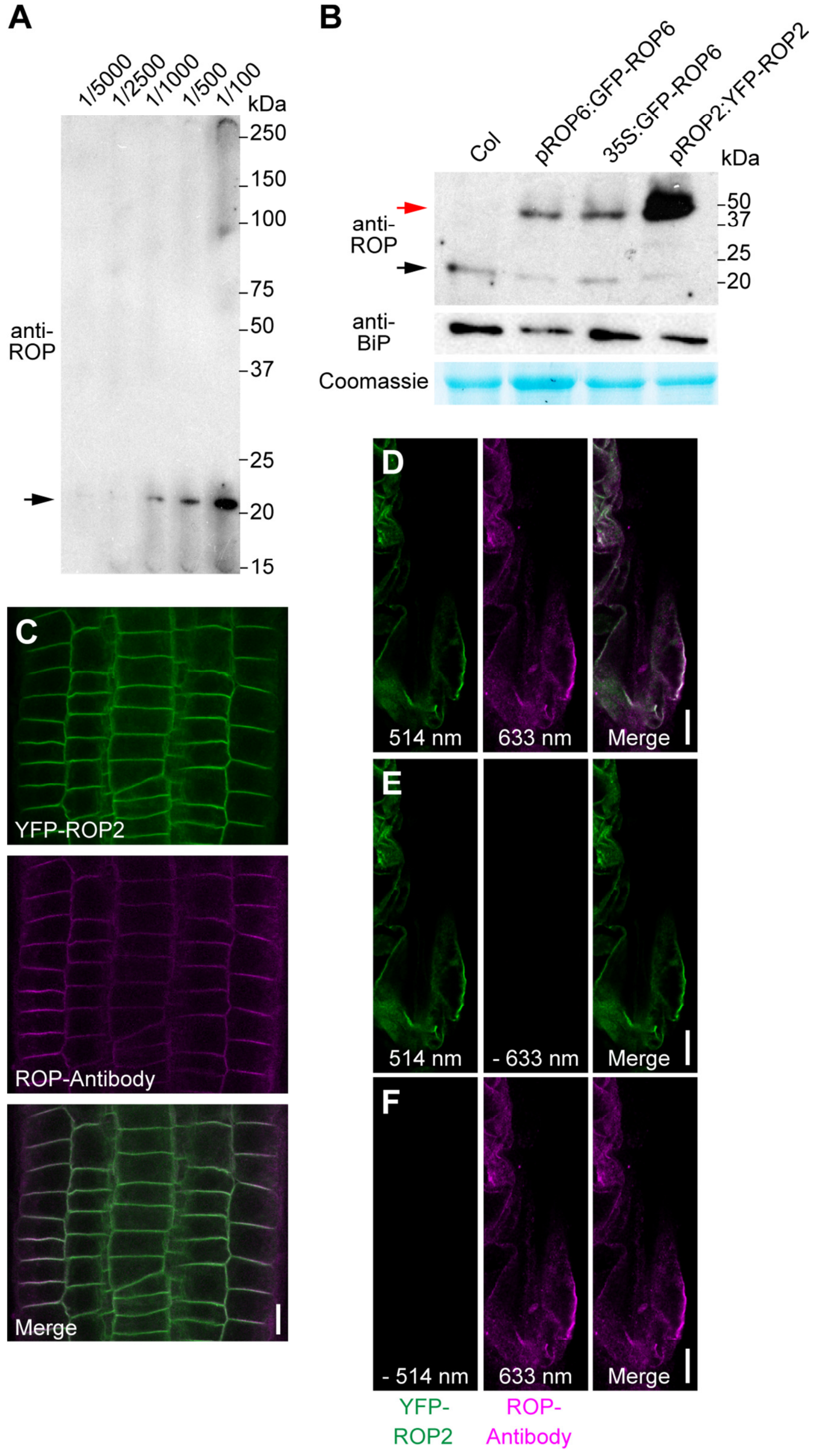

Fig. S5. Specificity of anti-AtROP antibodies. (A) Dilutions of affinity-purified anti-AtROP2 antibody from rabbit Vickie tested on total protein extracts (100 µg) from Col

seedlings. Arrow indicates the molecular weight of ROP (21 kDa). Note, optimum dilution for use of anti-AtROP2 antibody was 1/500. (B) Affinity-purified anti-AtROP2 antibody (1/500) was used on protein extracts (20 µg) from Col, *p35S:GFP-ROP6*, *pROP6:GFP-ROP6* and *pROP2:EYFP-ROP2*. Black arrow marks the expected size for the full-length ROP (21 kDa); red arrow marks the expected size of ROPs fused to GFP or YFP (48 kDa). Equal loading was tested using anti-BiP Western blot and Coomassie Blue staining. (C-G) Specificity of anti-AtROP2 from rabbit Calvin was tested on five-day-old (C-F) *pROP2:EYFP-ROP2* and (G) Col seedlings. (C) Note, strong co-labelling of EYFP-ROP2 (YFP-ROP2) (green) and anti-ROP2 detected with DyLight633 conjugated donkey anti-rabbit IgG antibody (magenta) is observed at the epidermal and cortical plasma membranes. (D) Co-localisation of EYFP-ROP2 (YFP-ROP2) (green) with anti-ROP2 (magenta) in an early forming hair bulge. (E,F) Absence of anti-ROP2 derived or EYFP-ROP2 signal in a forming bulge when only the 514 nm (E) or the 633 nm (F) lasers were activated, respectively. Scale bars: 10 µm.

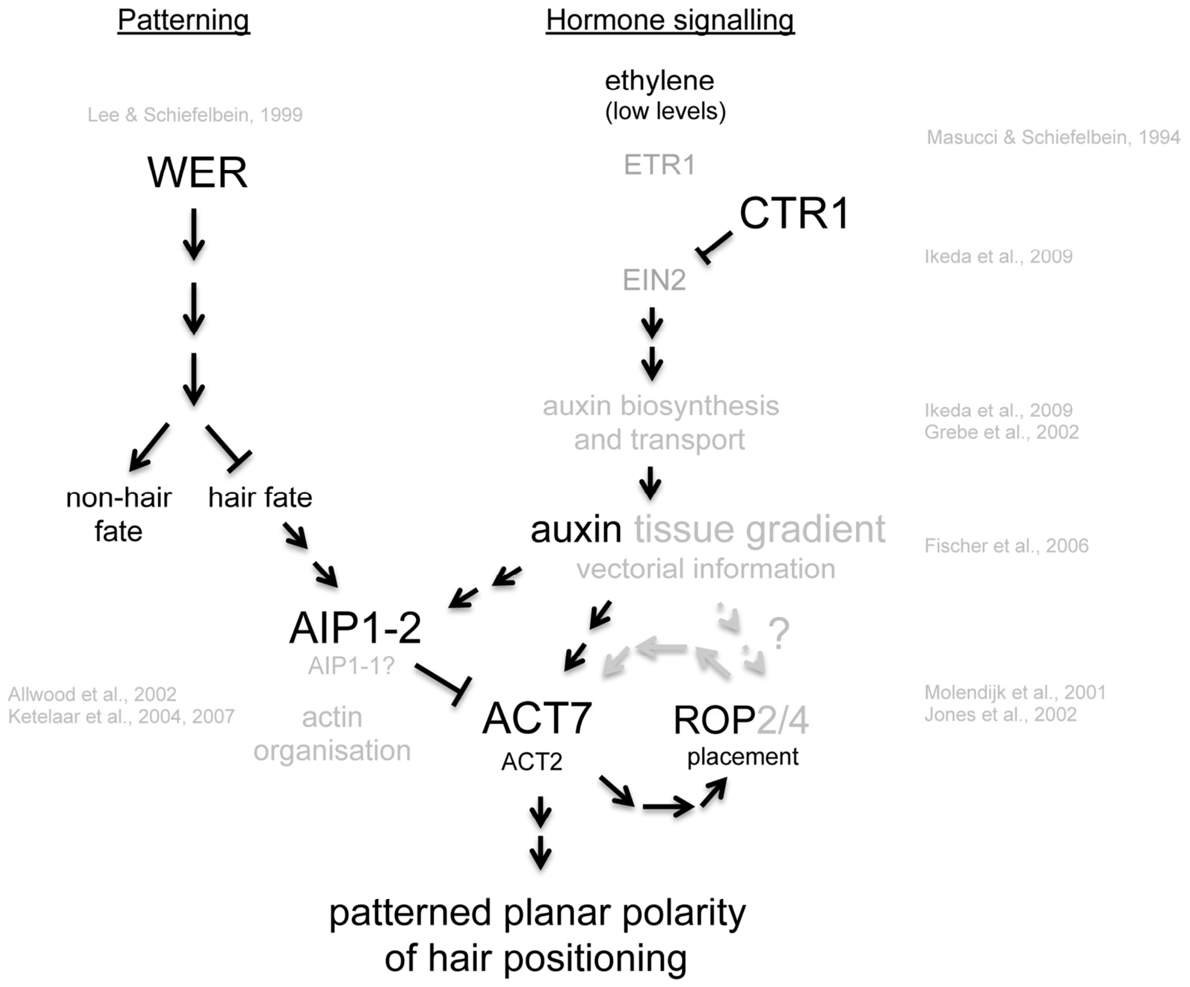

Fig. S6. A hypothetical model for AIP1-2 and actin action downstream of root hair patterning and ethylene signalling. WER specifies non-hair cell fate and thus represses hair cell fate (Lee and Schiefelbein, 1999). The present study reveals ectopic AIP1-2 expression in *wer* and in *ctr1* mutants placing AIP1-2 downstream of *WER*- and *CTR1*-mediated repression. Several additional components of the ethylene signalling pathway such as the ethylene receptor ETR1 (Masucci and Schiefelbein, 1994), EIN2 and genes acting in, or upstream of auxin biosynthesis have previously been shown to control planar polarity of ROP and hair positioning (Fischer et al., 2006; Ikeda et al., 2009). The present study shows that effects of *ctr1* mutation on planar polarity are repressed by *act2* and *act7* mutations and are

epistatic over *aip1-2* mutation suggesting *AIP1-2*, *ACT2* and *ACT7* action downstream of *CTR1*. Strikingly, positioning of the polar ROP patch relies on AIP1-2, ACT7 (this study) and CTR1 function (Ikeda et al., 2009). As to whether auxin also acts on ROP independently of ACT7 and AIP1-2 remains open (dotted line and question mark). Two ROPs likely involved in the process are ROP2 and ROP4 recognised by the antibodies employed detecting the polar ROP patch at the hair initiation site (this study; Molendijk et al., 2001) and localised to the hair initiation site by GFP fusions (Jones et al., 2002). Dominant-negative interference with ROP2/4 function or ROP2 overexpression cause defects in actin organisation and/or polar hair initiation (Molendijk et al., 2001; Jones et al., 2002). AIP1-2 directly interacts with actins such as ACT7 (this study) and most likely has a negative regulatory function, because RNA interference with both *AIP1-1* and *AIP1-2* causes strong actin bundling in root epidermal celles and defects in tip growth (Ketelaar et al., 2004; Ketelaar et al., 2007). Moreover, *Arabidopsis* AIP1-1 promotes actin depolymersing activity of lily pollen ADF on heterologous actin in vitro (Allwood et al., 2002). Note, single arrows or single inhibitory lines indicate direct interaction, two or more indicate non-direct interactions within the same pathway. Factors and interactions analysed in the current study are shown in black. Factors or interactions analysed in previous studies are shaded in grey and the relevant work is referenced in the model.
